# Supplementary material for: A novel G-quadruplex aptamer-based spike trimeric antigen test for the detection of SARS-CoV-2
Source: Mol Ther Nucleic Acids. 2021 Jun 24;26:321–32. doi: 10.1016/j.omtn.2021.06.014 (PMC8223116; doi:10.1016/j.omtn.2021.06.014)
Supplement: Document S1. Figures S1–S7 and Tables S1–S6 [file mmc1.pdf]

OMTN, Volume *26*

Supplemental information

A novel G-quadruplex aptamer-based spike trimeric antigen test for the detection of SARS-CoV-2

Ankit Gupta, Anjali Anand, Neha Jain, Sandeep Goswami, Anbalagan Anantharaj, Sharanabasava Patil, Rahul Singh, Amit Kumar, Tripti Shrivastava, Shinjini Bhatnagar, Guruprasad R. Medigeshi, Tarun Kumar Sharma, and DBT India Consortium for COVID-19 Research

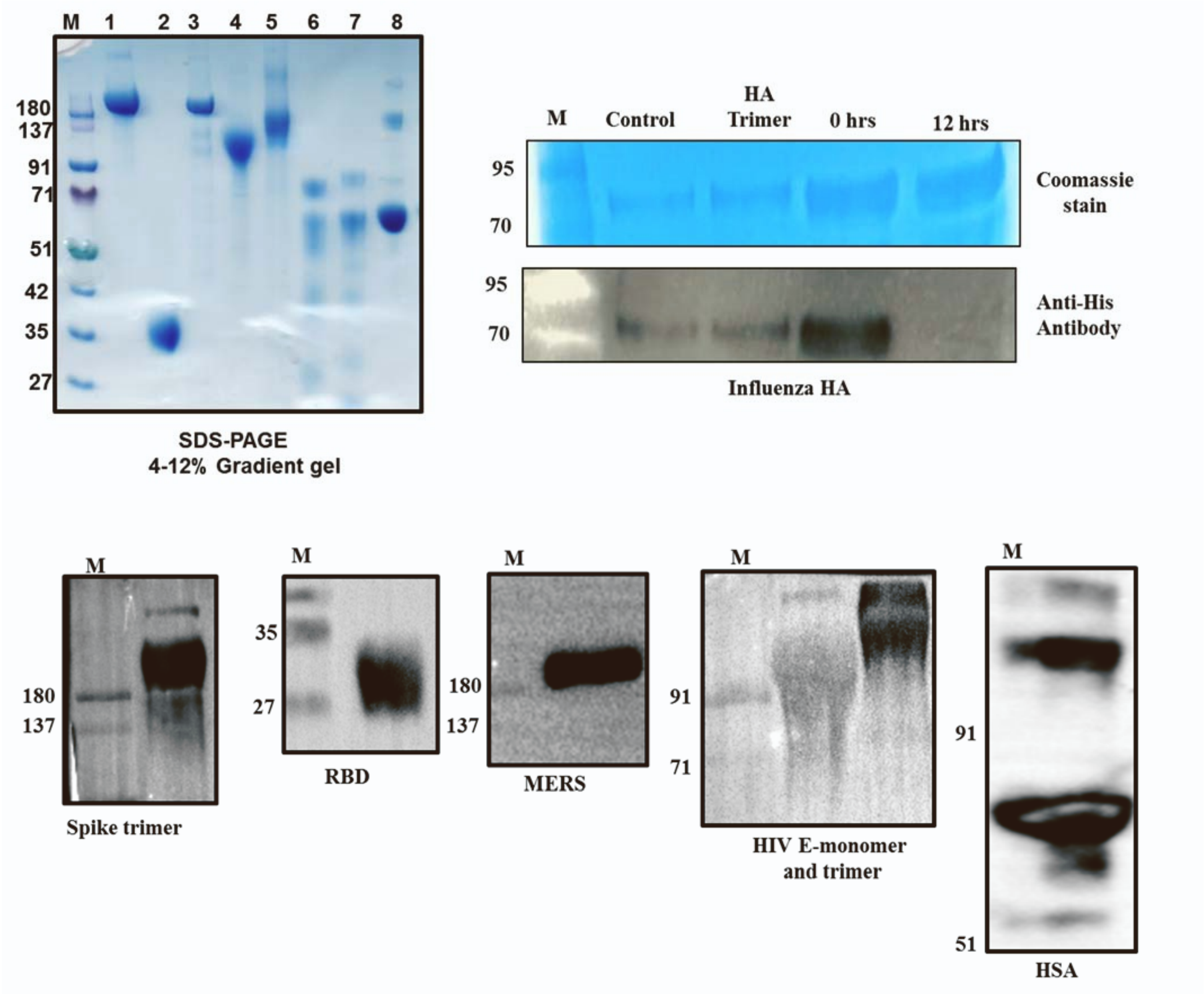

Figure S1. SDS-PAGE electrophoretogram of various proteins used in the study. lane 1 spike trimer protein (~194 kDa), lane 2 SARS spike-RBD (~ 32 kDa), lane 3 MERS FL-spike (~ 190 kDa), lane 4 HIV-E monomer (~ 95kDa), lane 5 HIV-E trimer (~ 130kDa), lane 6 Influenza HA monomer (~ 75 kDa), lane 7 Influenza HA trimer (~ 82 kDa) and lane 8 HSA (~ 66 kDa) on a gradient gel (4-12%). Western blot was developed using either the protein specific or anti-his antibody. M denotes the protein ladder in kDa.

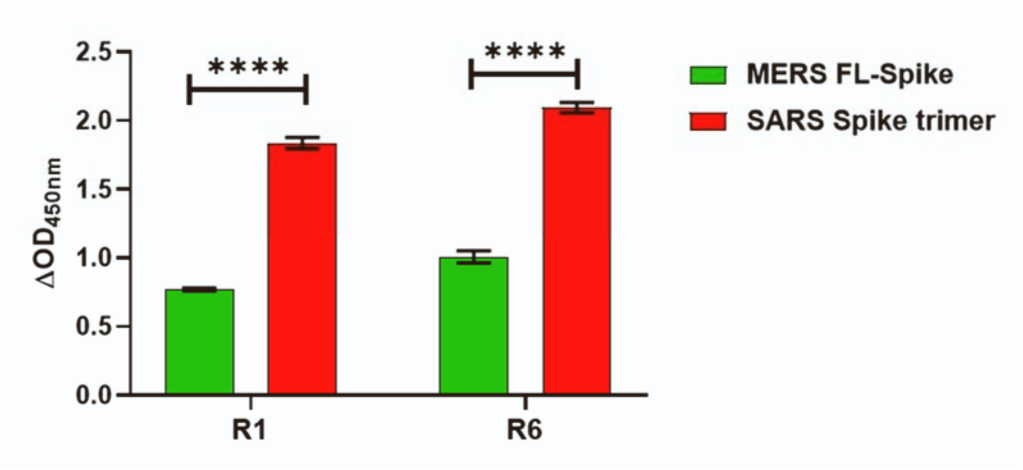

Figure S2. Comparing relative binding of round population (R1 and R6) enriched against spike trimer with MERS full length (FL)-spike. **** represent statistical significance at p value (***p < 0.0001). To compare the binding two-way ANOVA with multiple comparison was applied. Bars represent mean ± SD. ΔOD 450nm represents OD of wells with antigen subtracted OD of wells without antigen.

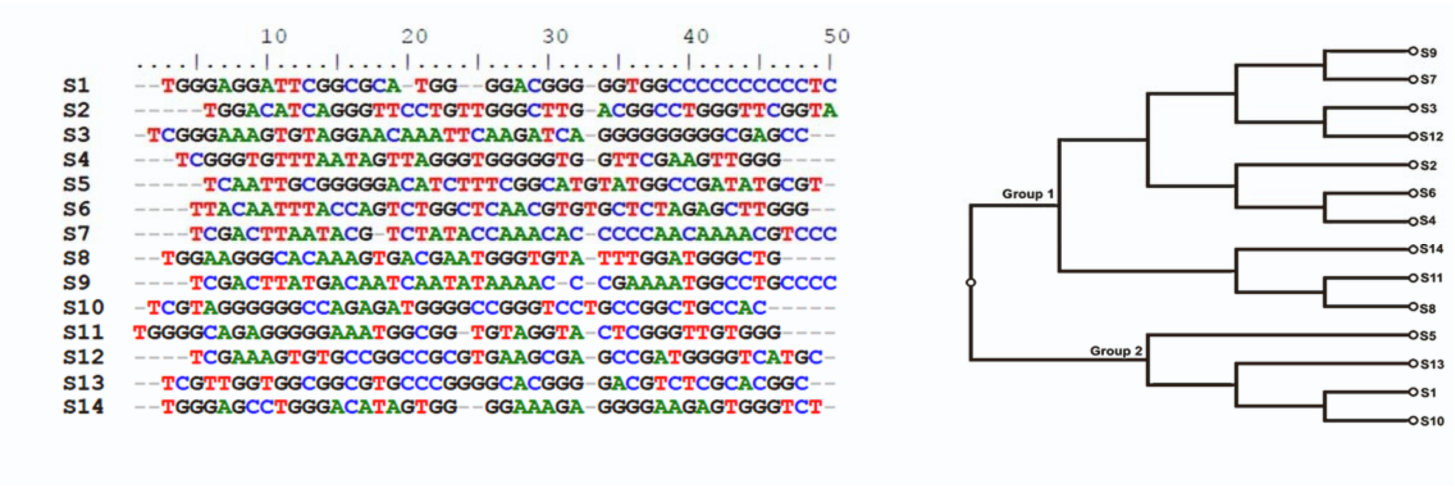

Figure S3. Multiple sequence alignment of 14 aptamer candidates targeting spike trimer antigen and ClustalW generated rooted phylogenetic tree analysis (UPGMA) for the aptamer candidates.

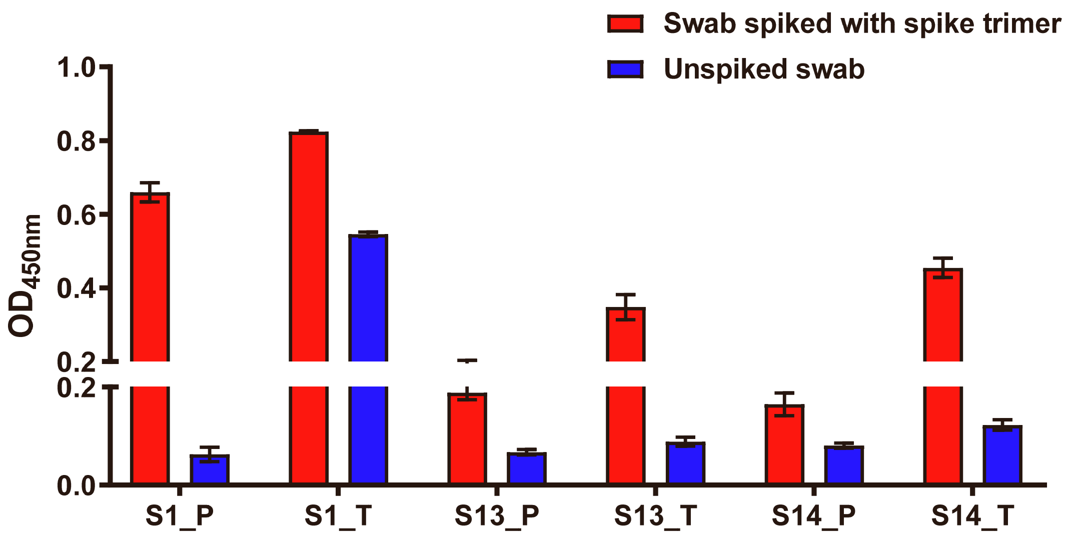

Figure S4. Comparison of binding performance parent (P) versus truncated (T) version of S1, S13 and S14 aptamer in viral transport media (VTM) spiked with antigen using ALISA. Bars represent mean ± SD.

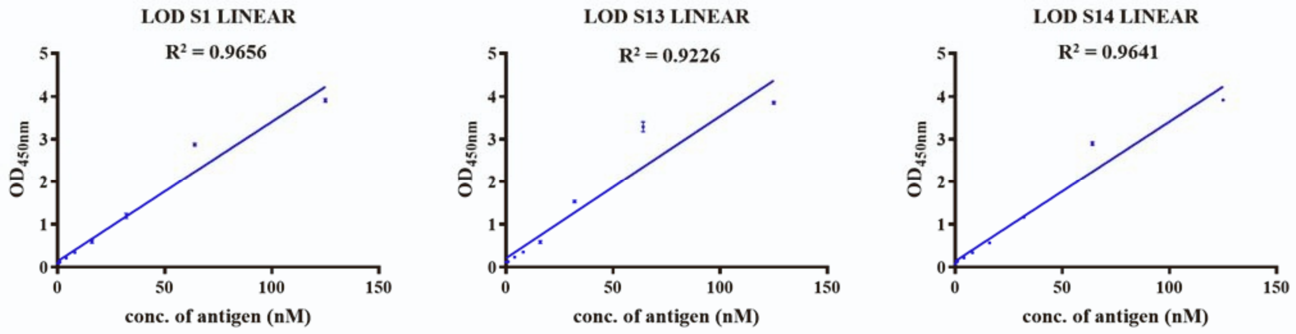

Figure S5

Figure S5. Linear dynamic range for 3 aptamers (S1, S13 and S14) from antigen concentration of 0.5-125 nM with a linearity regression coefficient (r^2) of >0.9226.

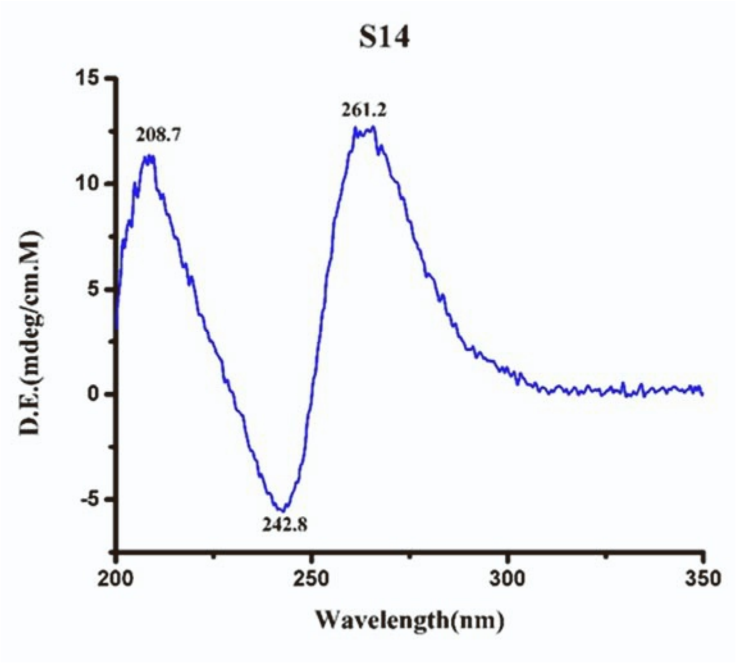

Figure S6. CD spectrum of S14 aptamer showing parallel G-quadruplex structure with signature peak at ~ 242 nm (negative) and ~ 261 nm (positive).

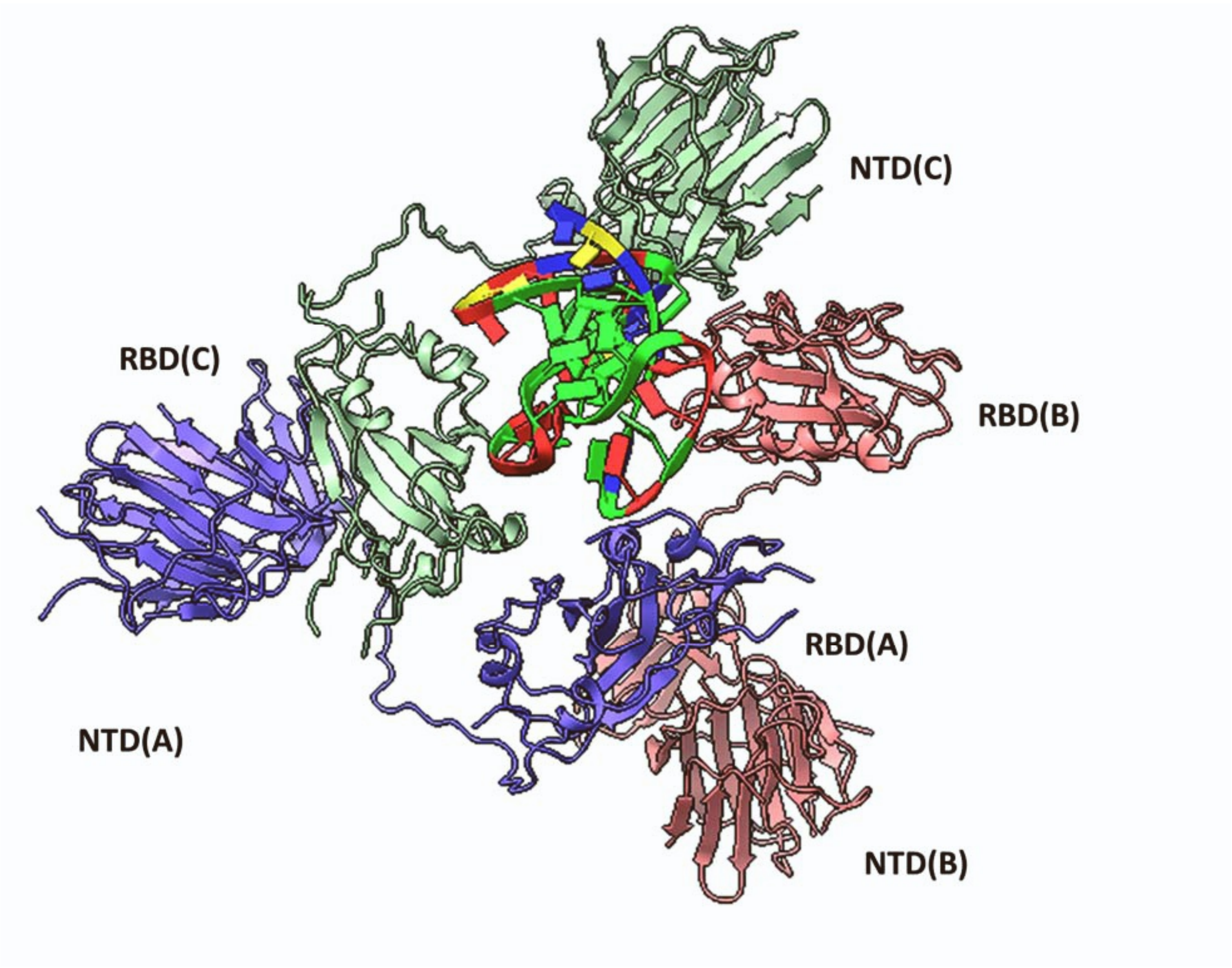

Figure S7. Top view of spike trimeric protein (showing only the NTD and RBD domains of all the three chains) in complex with the S14 aptamer. Aptamer is interacting with NTD(C) and RBD(B).

Table S1. MEME suite predicted motif in the aptamer sequences

Motif No.	Motif	E-value	% Occurence	Start Position in aptamers (nt no.)
1	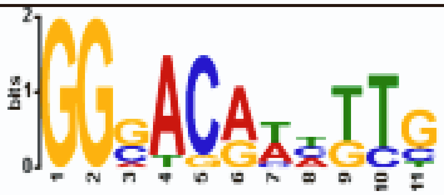	1.8e + 002	64.28	nt no. 10 in S14<br>nt no. 34 in S7<br>nt no. 7 in S8<br>nt no. 11 in S5<br>nt no. 14 in S3<br>nt no. 29 in S13<br>nt no. 3 in S12<br>nt no. 18 in S6<br>nt no. 2 in S1
2	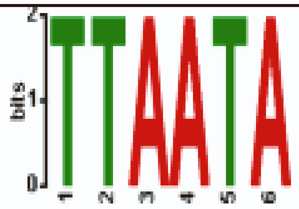	1.9e + 003	14.28	nt no. 6 in S7<br>nt no. 9 in S4
3	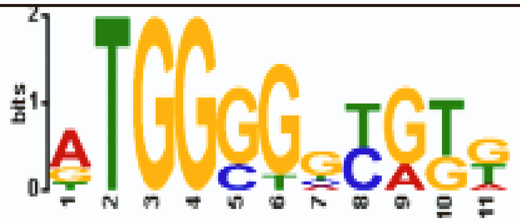	2.5e + 003	57.14	nt no. 23 in S7<br>nt no. 21 in S4<br>nt no. 17 in S11<br>nt no. 22 in S8<br>nt no. 33 in S12<br>nt no. 17 in S1<br>nt no. 7 in S13<br>nt no. 18 in S10
4	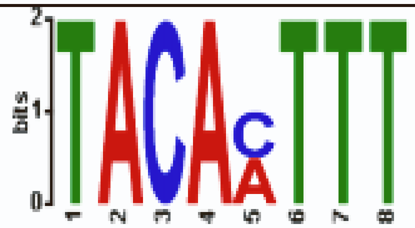	4.3e + 003	14.28	nt no. 2 in S6<br>nt no. 6 in S3
5	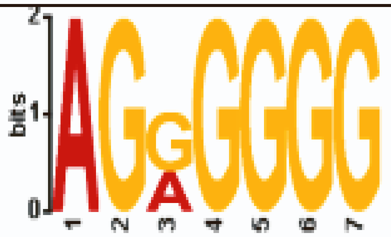	7.1e + 003	35.71	nt no. 5 in S10<br>nt no. 31 in S3<br>nt no. 37 in S1<br>nt no. 26 in S14<br>nt no. 7 in S11

Table S2. Clinical diagnostic performance of S14 aptamer in terms of sensitivity, specificity, positive predictive value, negative predictive value and accuracy.

Statistic	Value
Sensitivity	90.91%
Specificity	98.40%
Positive Predictive Value (*)	93.02%
Negative Predictive Value (*)	97.88%
Accuracy (*)	96.98%

Table S3. Putative overlapping G-quadruplex motifs in the S14 aptamer sequence. Red colored motifs with the highest G-score were used for structure prediction.

S.No.	Start Position	Length	QGRS	G-Score
1	2	31	GGGAGCCTGGGACATAGTGGGGAAAGAGGGG	70
2	2	30	GGGAGCCTGGGACATAGTGGGGAAAGAGGG	70
3	2	30	GGGAGCCTGGGACATAGTGGGGAAAGAGGG	69
4	2	31	GGGAGCCTGGGACATAGTGGGGAAAGAGGGG	69
5	2	40	GGGAGCCTGGGACATAGTGGGGAAAGAGGGGAAGAGTGGG	61
6	2	40	GGGAGCCTGGGACATAGTGGGGAAAGAGGGGAAGAGTGGG	61
7	2	40	GGGAGCCTGGGACATAGTGGGGAAAGAGGGGAAGAGTGGG	61
8	2	40	GGGAGCCTGGGACATAGTGGGGAAAGAGGGGAAGAGTGGG	60
9	2	40	GGGAGCCTGGGACATAGTGGGGAAAGAGGGGAAGAGTGGG	63
10	2	40	GGGAGCCTGGGACATAGTGGGGAAAGAGGGGAAGAGTGGG	63
11	2	40	GGGAGCCTGGGACATAGTGGGGAAAGAGGGGAAGAGTGGG	61
12	2	40	GGGAGCCTGGGACATAGTGGGGAAAGAGGGGAAGAGTGGG	62
**13**	**10**	**32**	**GGGACATAGTGGGGAAAGAGGGGAAGAGTGGG**	**71**
14	10	32	GGGACATAGTGGGGAAAGAGGGGAAGAGTGGG	70

Table S4. Table showing H-bond forming residues and distance between them.

S.No.	H-bonds forming residues	Length of H-bonds
1.	Pro463(B) – G32	2.5 Å
2.	Pro39 (C) – T1	3.4 Å
3.	Asp53 (C) – T1	2.1 Å
4	Asn234(C) - T16	2.3 Å
5.	Thr-376 (C) – G27	2.5 Å

Table S5. Mutant and control aptamer sequences used

Aptamer	Sequence	Bases
S14	TGGGAGCCTGGGACATAGTGGGGAAAGAGGGGAAGAGTGGGTCT	44
S14_M1	TGGGAGCCTGGGACA**C**AGTGGGGAAAGAGGGGAAGAGTGGGTCT	44
S14_M2	TGGGAGCCTG**T**GACATAGTG**T**GGAAAGAG**T**GGAAGAGTG**T**GTCT	44
S14_M3	TGGGAGCCTG**C**GACATAGTG**C**GGAAAGAG**C**GGAAGAGTG**C**GTCT	44
S14_M4	TGGGAGCCTGGGACATAGTG**T**GGAAAGAGGGGAAGAGTG**T**GTCT	44
Control aptamer	TTTTTTTTTTTTTTTTTTTTTTTTTTTTTTTTTTTTTTTTTTTTTTTT	48
G-quad control 1	GCAGAGAGAGAGAAAGTGAGAAGGGCGGGGGGGAGAGGGTGGGGG	45
G-quad control 2	GGGGGTGGTGGGGGGGATAGGGTTGGAGAGAGAGAGAGTGTGT	43

Table S6. Comparative study of available tests for antigen detection for SARS-CoV-2

TEST	TARGET	METHOD	LOD	SENSITIVITY (%)	SPECIFICITY (%)	SAMPLE	READ OUT	REF
RapiGEN	Not specified	LFA	-NA-	62	100	NP/OP Swab	Visual: colored bands	[1]
Liming bio	Not specified	LFA	-NA-	0	90	NP/OP Swab	Visual: colored bands	[1]
Savant	N protein	LFA	-NA-	16.7	100	Throat Swab	Visual: fluorescent bands (under UV light)	[1]
Bioeasy	Not specified	LFA	-NA-	85	100	NP/OP Swab	Visual: fluorescent readers	[1]
DETECTR	E and N gene	LFA with RT-Lamp and cas-12	10,000 copies/mL	95	100	NP/OP Swab	Visual	[2]
SHERLOCK	E, N, ORF1ab gene	LFA with RT-RPA and cas-13a	200 copies/mL	97	100	NP/OP Swab	Visual	[3]
Veritor (BD)	N protein	Chromatographic immunoassay	140 TCID50/swab	83.9	100	NP swab	Veritor Plus Analyzer	[4]
LumiraDx (LumiraDx)	N protein	Fluorescence immunoassay	32 TCID50/swab	97.6	96.6	NP swab	LumiraDx Instrument	[5]
COVID-19 Ag Respi-Strip	N protein	Dipstick LFA	5.4 pM	87.9	77.3	NP Swab	Visual	[6]

BinaxNow (Abbott)	N protein	LFA	22.5 TCID50/ swab	96.7	100	NP Swab	Visual	[7]
Xpert Xpress (Cepheid)	E, N genes	RT-PCR	250 copies/mL	97.8	95.6	NP, MT, OP swab	GeneXpert Dx	[8]
FELUDA	N and S genes	RT-RPA with LFA and cas-9	~ 10 copies	100	97	Saliva	Visual	[9]
Sandwich type COVID-19 Nucleocapsid Protein DNA Aptamers	N protein	Antibody-Aptamer based sandwich ELISA and LFA	1 ng/mL	-NA-	-NA-	sputum, serum, and urine	Visual	[10]
This Study	S protein	ALISA	2nM	91	98	NP/OP swabs	Visual and spectrophotometric	-NA-

LOD: Limit of Detection

LFA: Lateral Flow Assay

NP/OP: Nasopharyngeal/Oropharyngeal

ELISA: Enzyme Linked Immunosorbent Assay

S, E and N: Spike, Envelope and Nucelocapsid

References for Table S6:

1. Weitzel, T., Legarraga, P., Iruretagoyena, M., Pizarro, G., Araos, R., Munita, J.M., and Porte, L. Head-to-head comparison of four antigen-based rapid detection tests for the diagnosis of SARS-CoV-2 in respiratory samples.

2. Broughton, J.P., Deng, X., Yu, G., Fasching, C.L., Servellita, V., Singh, J., Miao, X., Streithorst, J.A., Granados, A., Sotomayor-Gonzalez, A., et al. CRISPR–Cas12-based detection of SARS-CoV-2.

3. Patchsung, M., Jantarug, K., Pattama, A., Aphicho, K., Suraritdechachai, S., Meesawat, P., Sappakhaw, K., Leelahakorn, N., Ruenkam, T., Wongsatit, T., et al. (2020). Clinical validation of a Cas13-based assay for the detection of SARS-CoV-2 RNA. Nat. Biomed. Eng. *4*, 1140–1149.

4. COVID-19 Rapid Antigen Testing | BD Veritor™ Plus System https://bdveritor.bd.com/en-us/rapid-antigen-testing/covid-19.

5. Connected diagnostics and diagnostic-led care solutions | LumiraDx https://www.lumiradx.com/us-en/.

6. Rodriguez-Palacios, A., Zeng, N., Berhane, Y., Vandenberg, O., Mertens, P., De Vos, N., Martiny, D., Jassoy, C., Mirazimi, A., Cuypers, L., et al. (2020). Development and Potential Usefulness of the COVID-19 Ag Respi-Strip Diagnostic Assay in a Pandemic Context. Front. Med. | www.frontiersin.org *1*, 225.

7. Abbott U.S. https://www.abbott.com/corpnewsroom.html.

8. Loeffelholz, M.J., Alland, D., Butler-Wu, S.M., Pandey, U., Perno, C.F., Nava, A., Carroll, K.C., Mostafa, H., Davies, E., Mcewan, A., et al. (2020). Multicenter Evaluation of the Cepheid Xpert Xpress SARS-CoV-2 Test.

9. Azhar, M., Phutela, R., Kumar, M., Ansari, A.H., Rauthan, R., Gulati, S., Sharma, N., Sinha, D., Sharma, S., Singh, S., et al. (2020). Rapid, accurate, nucleobase detection using FnCas9. medRxiv, 2020.09.13.20193581.

10. Zhang, L., Fang, X., Liu, X., Ou, H., Zhang, H., Wang, J., Li, Q., Cheng, H., Zhang, W., and Luo, Z. (2020). Discovery of sandwich type COVID-19 nucleocapsid protein DNA aptamers. Chem. Commun. *56*, 10235–10238.
