# Supplementary material for: A novel G-quadruplex aptamer-based spike trimeric antigen test for the detection of SARS-CoV-2
Source: Mol Ther Nucleic Acids. 2021 Jun 24;26:321–32. doi: 10.1016/j.omtn.2021.06.014 (PMC8223116; doi:10.1016/j.omtn.2021.06.014)
Supplement: Document S2. Article plus supplemental information [file mmc2.pdf]

Molecular Therapy
Nucleic Acids
Original Article

A novel G-quadruplex aptamer-based spike trimeric antigen test for the detection of SARS-CoV-2

Ankit Gupta,[1,6] Anjali Anand,[1,6] Neha Jain,[2] Sandeep Goswami,[3] Anbalagan Anantharaj,[4] Sharanabasava Patil,[4] Rahul Singh,[5] Amit Kumar,[2] Tripti Shrivastava,[3] Shinjini Bhatnagar,[4] Guruprasad R. Medigeshi,[4] Tarun Kumar Sharma,[1] and DBT India Consortium for COVID-19 Research

[1]Aptamer Technology and Diagnostics Laboratory, Multidisciplinary Clinical and Translational Research, Translational Health Science and Technology Institute, Faridabad, Haryana 121001, India; [2]Discipline of Biosciences and Biomedical Engineering, Indian Institute of Technology Indore, Simrol, Madhya Pradesh 453552, India; [3]Infection and Immunology, Translational Health Science & Technology Institute, NCR Biotech Science Cluster, Faridabad, Haryana 121001, India; [4]Translational Health Science and Technology Institute, Faridabad, Haryana 121001, India; [5]Raja Ramanna Centre for Advanced Technology, Indore, Madhya Pradesh 452013, India

The recent SARS-CoV-2 outbreak has been declared a global health emergency. It will take years to vaccinate the whole population to protect them from this deadly virus, hence the management of SARS-CoV-2 largely depends on the widespread availability of an accurate diagnostic test. Toward addressing the unmet need of a reliable diagnostic test in the current work by utilizing the power of Systematic Evolution of Ligands by EXponential enrichment, a 44-mer G-quadruplex-forming DNA aptamer against spike trimer antigen of SARS-CoV-2 was identified. The lead aptamer candidate (S14) was characterized thoroughly for its binding, selectivity, affinity, structure, and batch-to-batch variability by utilizing various biochemical, biophysical, and *in silico* techniques. S14 has demonstrated a low nanomolar K_D, confirming its tight binding to a spike antigen of SARS-CoV-2. S14 can detect as low as 2 nM of antigen. The clinical evaluation of S14 aptamer on nasopharyngeal swab specimens (n = 232) has displayed a highly discriminatory response between SARS-CoV-2 infected individuals from the non-infected one with a sensitivity and specificity of ~91% and 98%, respectively. Importantly, S14 aptamer-based test has evinced a comparable performance with that of RT-PCR-based assay. Altogether, this study established the utility of aptamer technology for the detection of SARS-CoV-2.

INTRODUCTION

In the past few decades, the human race has witnessed many pandemic situations where disease transmission occurs by microbial pathogens across communities and borders. Among these pathogens, viruses posed a major threat to humanity. In recent years, we have faced outbreaks of Severe Acute Respiratory Syndrome Coronavirus (SARS-CoV-2002 China), swine-origin pandemic (H1N1) influenza A virus (2009-Mexico), and the Middle East Respiratory Syndrome Coronavirus (MERS-CoV-2012 Saudi Arabia).[1–3] All these outbreaks have affected several thousand individuals. More recently, in 2019, the largest outbreak of this decade occured due to yet another coronavirus, Severe Acute Respiratory Syndrome Coronavirus-2 (SARS-CoV-2). This virus has stemmed from Wuhan, China and spread throughout the world.[4] Compared to SARS-CoV, there are many attributes including but not limited to aggressive disease transmission, high attack rates, minimal population immunity, contagiousness, and severity that makes SARS-CoV-2 distinct from SARS-CoV.[5] Due to its rapid transmission rate, SARS-CoV-2 was declared a global health emergency by the World Health Organization (WHO) in March 2020. As of May 21st, 2021, SARS-CoV-2 has accounted for an estimated active case of about ~15.8 million with ~3.4 million deaths globally[6] (https://www.worldometers.info/coronavirus).

Development of vaccines or drugs against novel viruses during a pandemic outbreak is very challenging as the discovery of potent lead molecules or vaccine itself is a time-consuming and tedious process as it involves various phases of clinical trials.[7] In early 2021, many vaccine candidates were approved and rolled out, and yet it will take some years to vaccinate the whole population to protect them from this deadly virus.[8,9] However, rapid screening/detection of the population for the infection may help with containing the disease. The majority of these *in vitro* screening and detection assays rely upon real-time polymerase chain reaction (PCR) or antibodies.[10] The former requires a sophisticated machine, expensive probes, and a highly trained manpower, while antibodies have to be generated in an animal and require several booster doses.[11] Overall, antibody generation is a several month process. Further, they are prone to batch-to-batch

Received 10 November 2020; accepted 9 June 2021;
https://doi.org/10.1016/j.omtn.2021.06.014.

[6]These authors contributed equally

Correspondence: Tarun Kumar Sharma, Aptamer Technology and Diagnostics Laboratory, Multidisciplinary Clinical and Translational Research, Translational Health Science and Technology Institute, Faridabad, Haryana 121001, India.
E-mail: tarun@thsti.res.in

This is an open access article under the CC BY license (http://creativecommons.org/licenses/by/4.0/).

variation and thus have suffered from reproducibility crisis. In 2008, a validation survey conducted by the Human Protein Atlas evaluated >5,000 commercially available antibodies from ~50 different vendors using western blot and immunohistochemistry methods. Their data strongly indicate the reproducibility crisis of antibodies as ~50% of antibodies could not be validated successfully.[12] In a recent report, this problem is elegantly described where researchers have used 16 commercially available antibodies from 7 different sources.[13] Out of these 16 antibodies, only one worked in immunofluorescence assay while the other two worked for western blotting experiments. Considering such problems associated with antibodies along with the outbreak of a novel virus like SARS-CoV-2, it is difficult to rely upon antigen detection assays using antibodies. Further, even if we are lucky, getting a good antibody may take several months.[14] Alternatively, detection of immunoglobulin M (IgM) and IgG in patient sera using recombinant viral antigens can be a reasonable and pragmatic approach, but it suffers from poor sensitivity as seroconversion takes several days to weeks as it may vary from patient to patient.[15] Another limitation is that this approach cannot work for immunocompromised and hemodialyzed patients.[13] Therefore, it is imperative to develop an aptamer-based antigen detection assay as nucleic acid aptamers can bind to its target with high affinity and selectivity without/negligible batch-to-batch variation.[16] Further, they are amenable to various sensing platforms present in centralized laboratory to decentralized point-of-care (POC) devices used for diagnostic applications.[17,18] In recent years, aptamer technology has emerged as a rapid method to generate high-quality nucleic acid binders or chemical antibodies against a wide variety of analytes ranging from small molecules to protein (antigen) to whole prokaryotic or eukaryotic cell in few weeks to a month time.[19] Further, ease in functionalization with various moieties like fluorophore, biotin, redox label, and nanomaterials, etc. makes them an attractive tool for diagnostic applications.[20] Moreover, being a synthetic molecule, aptamers can easily be synthesized chemically using automated DNA synthesizer that allows their rapid large-scale production with almost no batch-to-batch variation.[21] To bridge the existing diagnostic gap for SARS-CoV-2, we herein report the identification, characterization, and diagnostic application of spike trimer binding aptamers. The best performing aptamer candidate S14 evinced its high diagnostic utility to be used as a frontline laboratory-based assay for the detection of SARS-CoV-2 antigen.

RESULTS

We herein report the spike trimer antigen-specific aptamer development using talon bead-based SELEX, post-SELEX optimization, *in silico*, and biophysical characterization. Finally, the application of best performing aptamer candidate was demonstrated to detect the presence of spike trimer antigen in clinical specimens, nasopharyngeal swabs (NP, n = 232).

SELEX-based identification of DNA aptamers against spike trimer protein of SARS-CoV-2

For aptamer selection, prefusion spike trimeric protein (~194 kDa) was used as a target (Figure S1). To obtain highly specific and affine DNA aptamers against spike protein, we followed a cobalt resin (Talon)-based SELEX strategy that involved the following steps: (1) incubation of aptamer libraries with cobalt resin to remove resin binders (negative selection), (2) incubation of unbound DNA sequences with spike protein in solution (binding buffer, BB), (3) addition of cobalt resin to capture aptamers-spike complex on resin, (4) elution and enrichment of spike binding aptamers, followed by single-stranded DNA (ssDNA) separation for next round and (5) finally, at the end of SELEX (six rounds) enriched aptamer pool was cloned and sent for sequencing (Figure 1A).

After six rounds of cobalt resin-based SELEX, the aptamer pool that was archived at the end of each round (round 1–6) were biotinylated by incorporating the 5′ biotin primer at PCR stage as described previously.[22] The strand separation of PCR product was achieved on Urea-PAGE, and 5′ biotinylated strand was eluted and used to assess its binding to spike trimer antigen in an aptamer-linked immobilized sorbent assay (ALISA, Figure 1B) to monitor the enrichment of aptamers over successive rounds of SELEX. Interestingly, from round one onward, aptamer binding to spike antigen appeared to be saturated. Binding of enriched library (R6) was compared with unenriched DNA random library (DRL). As shown in Figure 1C enriched library evinced ~2.5-fold higher binding compared to its unenriched counterpart. To ascertain the specificity of enriched aptamer library, we also assessed its binding against a similar protein, MERS full-length (FL)-spike (~190 kDa). It is evident from the Figure S2 that despite of having very high similarity between spike of MERS and SARS-CoV-2, aptamer pool of R1 and R6 are able to discriminate between these two proteins. Interestingly, both populations demonstrated the similar binding pattern (Figure S2). Based on these observations, enriched library of round 6 were cloned and sent for sequencing. Based on the DNA sequencing data, 14 representative aptamer candidates were selected and subjected to multiple sequence alignment (MSA). MSA-derived phylogenetic tree (Figure S3) of these aptamer sequences clearly indicates that all aptamers are clustered into two groups, with group 1 being the largest one as ~71% aptamer candidates are clustered together in this group. Further, for the identification of conserved motifs among selected aptamer candidates, they were subjected to Multiple Em for Motif Elicitation (MEME), an online tool (http://meme-suite.org). Results for the same were analyzed on the basis of low E value, among the 14 selected aptamer candidates a total 5 motifs ranging from 6 nt to 15 nt in length was observed. Motif-1 was highly prevalent with an occurrence among nine aptamer candidates out of 14 aptamers (~65%, Table S1).

The selected 14 aptamer candidates (Table 1; Figure S3) were subjected to ALISA to assess their ability to detect spike antigen. These aptamer candidates contain primer binding domain (PBD), which is unlikely to play any significant role in aptamer binding to its cognate target.[23] Therefore, we have also compared the binding of these aptamer candidates with their truncated counterpart, where the PBD was deleted from each aptamer. Interestingly, the binding of the truncated version of each aptamer was comparable to that of its respective parent aptamer (Figure 1D). Based on this data, three

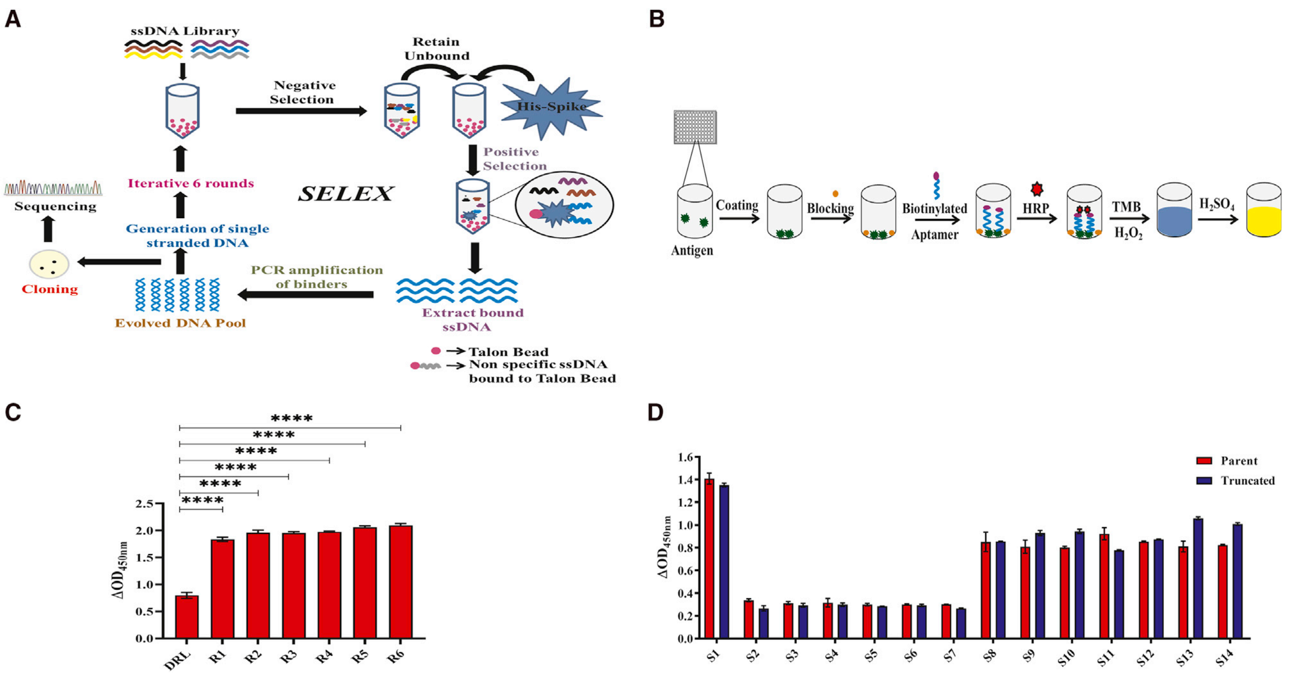

Figure 1. Detailed schematic approach for SELEX of spike trimer

(A) DNA libraries were incubated with talon beads for negative selection. Unbound DNA was retained and added to spike trimer immobilized on to the talon-beads for positive selection. Then binders were eluted and enriched through PCR. PCR amplicon was made single-stranded for the next round of SELEX. After six rounds of SELEX, enriched aptamer pool was cloned and sequenced. (B) Schema of aptamer-linked immobilized sorbent assay (ALISA) for the detection of spike trimer of SARS-CoV-2. Antigen was coated in 96-well plates followed by blocking of marginal sites. Blocked wells were incubated with biotinylated aptamer followed by washing to remove the unbound aptamer. Then wells were incubated with HRP-conjugate. Using TMB-H_2O_2 solution aptamer and spike antigen interaction was monitored in terms of colorimetric response and the reaction was stopped by H_2SO_4. (C) Comparing the relative binding of unenriched DRL pool versus enriched aptamer pools (R1–R6) with 125 ng amount of spike trimer using ALISA. (D) Effect of truncation of aptamers on its binding to spike antigen in comparison to their respective parent counterpart. ΔOD represents the OD of wells with antigen subtracted with OD of wells without antigen. To compare the binding, we applied one-way ANOVA with multiple comparison. **** represent statistical significance at p value (***$p < 0.0001$). Error bars represent mean ± SD.

aptamers (S1, S13, and S14) that were showing $\Delta OD_{450nm} > 1.0$ were selected for subsequent studies. Next to this, we have also assessed the binding of the parent (75/80/81-mer) and truncated version (44/45-mer) of these three aptamers in the viral transport media (VTM) background. Based on the outcome of this experiment (Figure S4), the parent aptamer of S1 (75-mer), and truncated aptamer of S13 (45-mer) and S14 (44-mer) were selected for further studies.

Assessment of cross-reactivity of aptamers

The top three aptamer candidates (S1, S13, and S14) selected for their high binding aptitude were assessed for their ability to selectively recognize spike trimer protein. The binding of these aptamer candidates were evaluated with various viral proteins, namely human immunodeficiency virus envelope (HIV-E) protein (both monomer and trimer form), monomeric and trimeric (fused with T4 fibritin motif) hemagglutinin (HA) protein (a spike-like protein) of influenza virus, and FL spike protein of MERS coronavirus. In addition to this, we have also used human serum albumin (HSA) and receptor binding domain (RBD) of the spike protein of SARS-CoV-2 to establish the selectivity of these aptamer candidates. The purity of these proteins was assessed using SDS-PAGE and western blotting (Figure S1). Interestingly, all aptamer evinced a highly selective response in the presence of spike trimer protein, and no significant cross-reactivity was observed with other viral proteins (Figures 2A–2C). Further, none of the aptamers showed any binding to spike RBD indicating that aptamers are binding to their respective aptatopes located on the spike trimeric protein other than the RBD portion. Taking together, these results unequivocally demonstrated the high selectivity of these aptamers for spike protein.

Analytical performance of selected aptamer candidates

To determine the low-end detection limit of selected aptamer candidates, we measured the response of each aptamer in the presence of a range of spike trimer proteins (2–500 nM). It is evident from Figures 2D–2F that all aptamers can detect as low as 2 nM of the spike antigen. To establish a dynamic linear range, we plotted the aptamer response OD450 nm as a function of spike antigen concentration. All three aptamers demonstrated a linear dynamic range from the concentration of 0.5 to 125 nM with a linearity regression coefficient (r^2) of > 0.9226 (Figure S5). Next, we assessed the binding of these three aptamers along with a control oligo in RT-PCR positive ($n = 5$) and negative ($n = 10$) NP swab samples. This data clearly shows

Table 1. Truncated sequences for all selected aptamers

Aptamer	Sequence 5′-3′	Bases
S1	TGGGAGGATTCGGCGCATGGGGA CGGGGGTGGCCCCCCCCCCTC	44
S2	TGGACATCAGGGTTCCTGTTGGG CTTGACGGCCTGGGTTCGGTA	40
S3	TCGGGAAAGTGTAGGAACAAATTC AAGATCAGGGGGGGGGCGAGCC	46
S4	TCGGGTGTTTAATAGTTAGGGTGG GGGTGGTTCGAAGTTGGG	42
S5	TCAATTGCGGGGGACATCTTTCGG CATGTATGGCCGATATGCGT	44
S6	TTACAATTTACCAGTCTGGCTCAAC GTGTGCTCTAGAGCTTGGG	44
S7	TCGACTTAATACGTCTATACCAAACA CCCCCAACAAAACGTCCC	44
S8	TGGAAGGGCACAAAGTGACGAATGG GTGTATTTGGATGGGCTG	43
S9	TCGACTTATGACAATCAATATAAAA CCCGAAAATGGCCTGCCCC	44
S10	TCGTAGGGGGGCCAGAGATGGGGC CGGGTCCTGCCGGCTGCCAC	44
S11	TGGGGCAGAGGGGGAAATGGCGG TGTAGGTACTCGGGTTGTGGG	44
S12	TCGAAAGTGTGCCGGCCGCGTGA AGCGAGCCGATGGGGTCATGC	44
S13	TCGTTGGTGGCGGCGTGCCCGGG GCACGGGGACGTCTCGCACGGC	45
S14	TGGGAGCCTGGGACATAGTGGGGA AAGAGGGGAAGAGTGGGTCT	44

that S13 and control oligo were not as efficient as S1 and S14 (Figure 3A) in detecting the spike trimer antigen in the clinical specimens. Hence, aptamer S1 and S14 were selected for further binding studies.

Bio-layer interferometry (BLI)

To determine the strength of aptamer and spike trimer antigen interaction, we have determined the dissociation constant of selected aptamer (S1 and S14) candidates using BLI. It is a label-free technology to measure biomolecular interactions in real-time. The 5′ biotinylated aptamer was immobilized on a streptavidin (SA) sensor, and spike trimer protein at various concentrations (7.4–600 nM) was used as an analyte. The aptamer evinced a very tight binding with the spike protein, which is characterized by a K_D in low nanomolar range using an appropriate (1:1) ligand binding model (Figure 3B). The aptamer S14 evinced ~3-fold higher affinity (K_D = 21.8 nM) then S1 (K_D = 68.9 nM). Considering the higher affinity of aptamer S14, it was selected for further evaluation.

Assessment of batch-to-batch variation

Being a synthetic molecule, aptamers are considered to have an additional advantage over their antibody counterpart viz negligible batch-to-batch variation. However, in most of the aptamer-based diagnostic studies, data on the batch-to-batch variation of aptamers are missing.[4,24–27] Therefore, in the current investigation, we have assessed the seven different batches of aptamer S14 obtained from three different manufacturers to evaluate their batch-to-batch variation (Figure 3C). Interestingly, no significant variation was observed in terms of aptamer binding to its cognate target. This data strongly indicates the reproducibility of aptamer performance regardless of its batches.

Aptamer-based detection of spike trimeric antigen in NP swab samples

To establish the real clinical utility of lead aptamer candidate (S14), its diagnostic potential was assessed in blinded manner on 232 NP swab samples obtained from COVID-19 suspects. In parallel, a gold standard test (real-time PCR) was also performed on these samples. To achieve a ~98% specificity, we derived a cut-off (0.104) using a receiver operating characteristic (ROC) curve. The ROC curve exhibited an area under the curve equivalent to 0.99, indicating the robustness of the assay.[28,29] Based on the ROC derived cut-off, the performance of the assay was determined. The aptamer S14 demonstrated ~91% sensitivity with ~98% specificity using real-time PCR as gold standards. These results clearly indicated the translational value of the aptamer candidate for the detection of SARS-CoV-2 infection (Figure 3D; Table S2).

Structural characterization of aptamers and its interaction with the spike protein

In order to gain structural insight of S14 aptamer, its secondary structure was predicted by RNAfold server using DNA parameters and G-quadruplex prediction options (Figure 4A).[30] The minimum free energy (MFE) predicted secondary structure of S14 contains a stem-loop-like structure with guanine -cytosine/guanine-thymine (GC/GT)-rich sequence forming G-quadruplex in the loop region. Interestingly, aptamer S14 has high guanine content; therefore, its G-quadruplex-forming ability was also checked using QGRS mapper and G-score was determined. QGRS mapper results indicated that S14 aptamer can form multiple overlapping G-quadruplex motifs (Table S3). To substantiate these observations, we subjected S14 aptamer to circular dichroism (CD). The CD spectrum of S14 aptamer showed a negative peak at ~242 nm and positive peak at ~261 nm (Figure S6), signifying a parallel G-quadruplex DNA structure.[31,32] Also, to assess the effect of various salts on the binding of S14 aptamer, a variety of buffers were prepared and CD spectra were collected for each buffer condition. The CD spectra highlighted the formation of G-quadruplex topologies in all the buffers including phosphate-buffered saline (PBS) alone (without any cation, Figure 4B). Interestingly, in presence of potassium and magnesium ions (buffer 6) G-quadruplex stabilization was observed and is clearly evident as increase in CD intensity. These observations are in concordance with previously published reports.[33,34]

To check the interaction of S14 with the spike protein, we analyzed CD spectra by using one concentration of the S14 aptamer with the increasing concentration of spike protein. The CD spectra of S14 showed a decrease in the negative peak with an increase in the positive

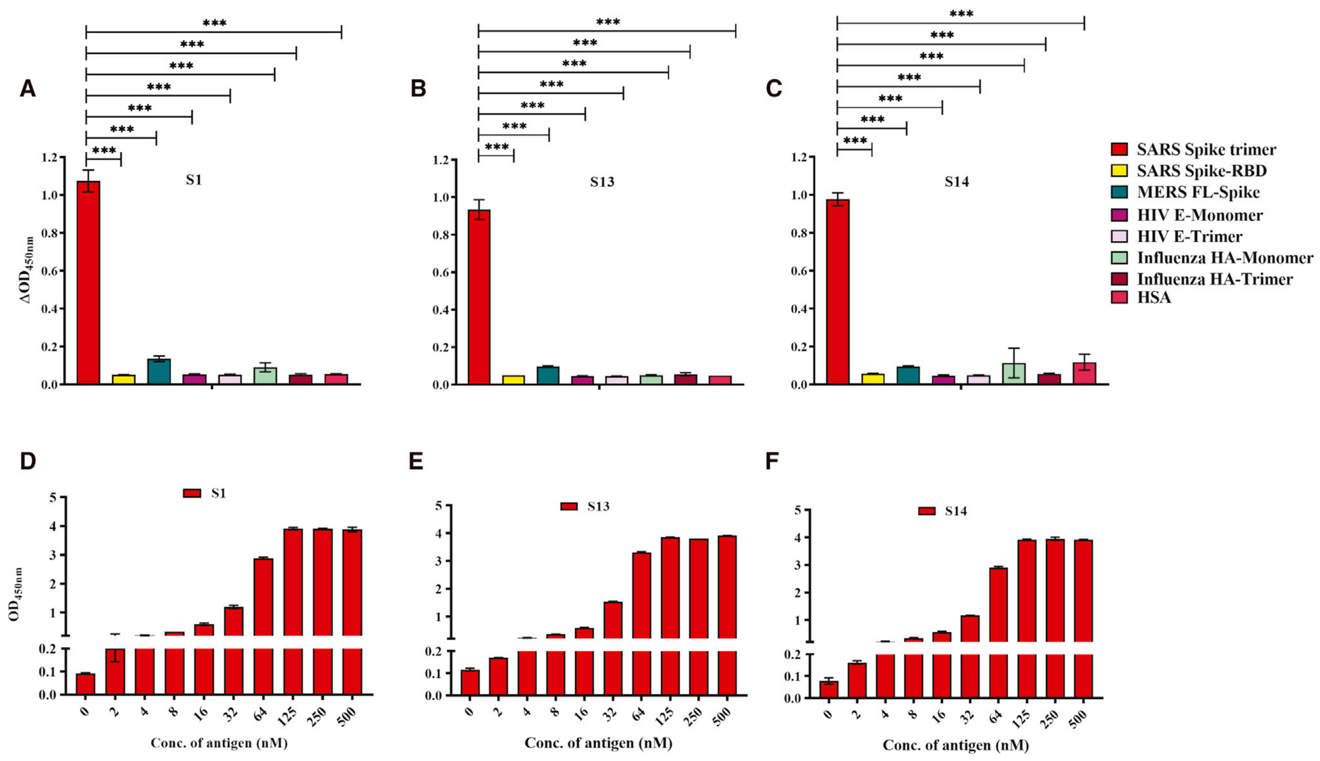

Figure 2. Assessment of cross-reactivity and LOD for 3 best-performing aptamers

(A) S1, (B) S13, and (C) S14 with SARS spike trimer, SARS spike-RBD, MERS FL-spike, HIV-E monomer, HIV-E trimer, influenza HA-monomer, influenza HA-trimer, and HSA. (D–F) Shown are LOD of all three aptamers with recombinant spike trimer (0–500 nM). *** represent statistical significance at p value (***$p < 0.0001$). To compare the binding, we applied two-way ANOVA ($n = 2$) with multiple comparison. Error bars represent mean ± SD.

peaks (Figure 4C). This change in CD spectra further affirmed the stable interaction of S14 with the spike protein of SARS-CoV-2 (Figure 4C).

To gain further insights into the molecular interaction of S14 with the spike trimeric protein, we used the G-quadruplex-forming motif with the highest G-score of 71 in the QGRS mapper prediction results for the S14 aptamer structure prediction (Figure 4D). The complete structure of the aptamer based on the parallel intra-molecular G-quadruplex structural motif was constructed and used for molecular docking analysis (Figure 5). Interaction analysis revealed the binding of S14 aptamer in the S1 region of the spike trimeric protein. Specifically, S14 binds between the N-terminal domain (NTD) of chain C and RBD domain of chain B (PDB ID: 6VYB). A total of five H-bonds (Table S4) were formed between the S14 aptamer and the spike trimeric protein (Figure 6A). G27 and G32 of the G-quadruplex loop regions interacted with the Thr376 (C) and Pro-463 (B), respectively, while T16 formed an H-bond with Asn23 (C), and T1 formed two H-bonds, one each with Pro39 (C) and Asp53 (C). The formation of four hydrogen bonds with the NTD domain (Asn23, Pro39, Asp53, and Thr376) of the C chain depicted the preferential interaction of S14 aptamer with the NTD rather than the RBD of the spike protein (Figure S7).

Effect of mutation on S14 aptamer

To investigate the impact of deletion of critical nucleotides as predicted by molecular modeling and docking studies, we have created four mutants by mutating the parent aptamer S14. As T-16 was one of the most interacting residues, it was deleted to generate S14_M1 mutant. Further, to check whether the disruption of G-quadruplex structure affected the binding of S14 with the spike protein, we generated three mutants by substituting the central guanine of G-tracts with thymine (S14_G4_M2), cytosine (S14_G4_M3), and G substituted with T in only the two G-tracts keeping intact the other two (S14_G4_M4; Table S5). On the CD spectral analysis of these mutants, signature pattern of parallel G-quadruplex was depicted by the S14_M4, while other three mutants (S14_M2, S14_M3, and S14_M4) showed a typical CD spectrum of a B-DNA (negative peak ~250 nm and positive peak ~275 nm). For the sake of comparison, two control oligo having parallel G-quadruplex structure were also subjected to CD analysis (Figure 6B).[33] Interestingly, on comparing the binding of these mutants with the spike protein, we observed ~31%, ~25%, and ~13% reduction in binding of S14_M3, S14_M4, and S14_M2 respectively highlighting the role of G-quadruplex formation in spike protein interaction. On comparing the S14_M1 mutant's binding with the S14 aptamer, the observed ~13% reduction.

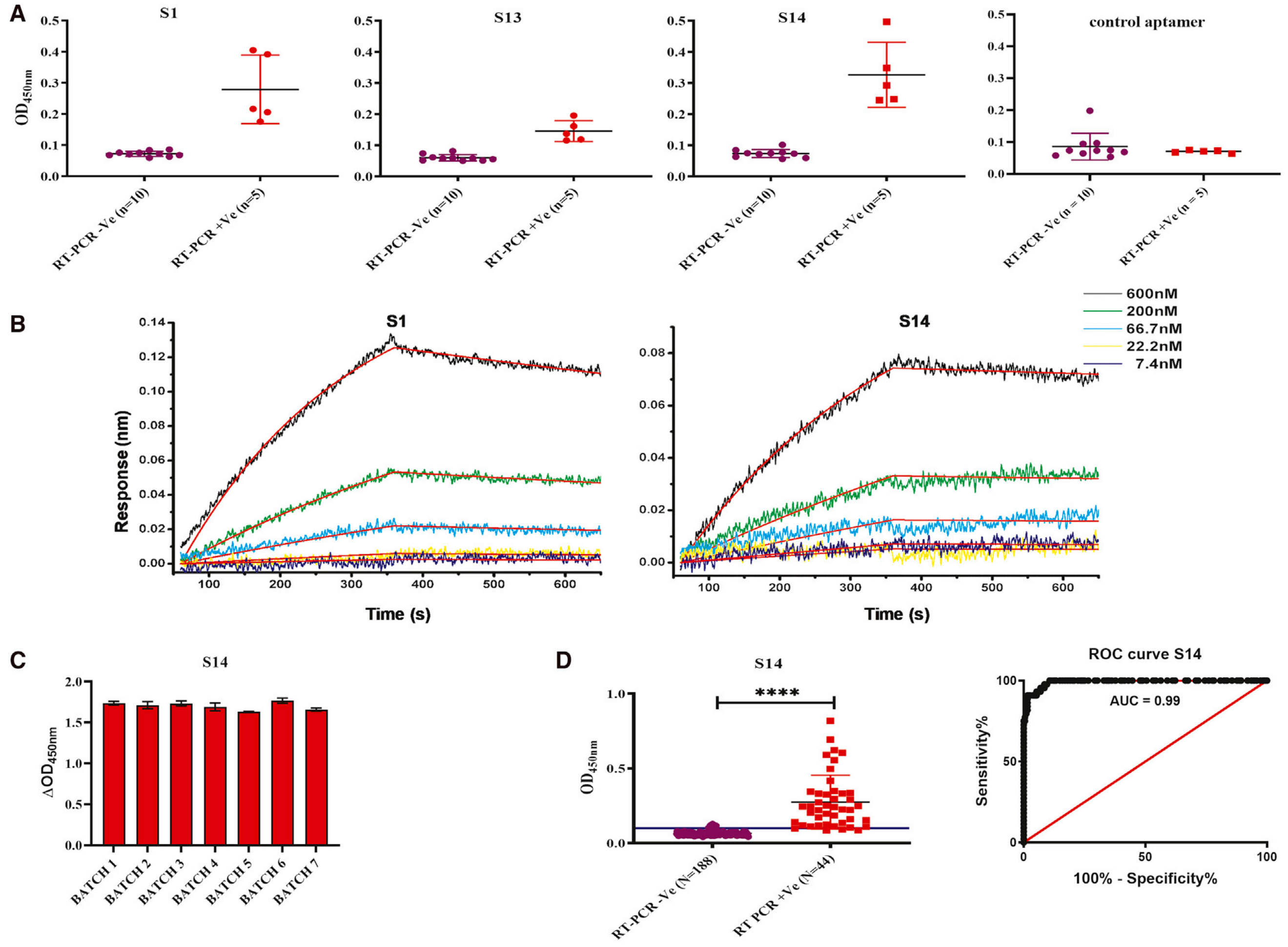

Figure 3. Binding efficiency, kinetics, batch-to-batch variability, and detection efficiency of aptamers

(A) S1, S13, S14, and control aptamer for the detection of spike antigen in RT-PCR negative (n = 10) and positive (n = 5) by ALISA in NP swab samples. (B) Binding kinetics of aptamer S1 and S14 with spike antigen at 3-fold dilution for the determination of binding affinity (K_D) using Bio-Layer Interferometry (BLI). (C) Determination of batch-to-batch variability of aptamer S14 by ALISA, seven different batches were tested. (D) Detection efficiency of S14 aptamer in RT-PCR negative (n = 188) and positive (n = 44) NP swab samples by ALISA. Receiver Operating Characteristic (ROC) curve showing specificity (~98%) and sensitivity (~91%) of the test. To compare the binding, unpaired t test was applied. **** represent statistical significance at p value (****p < 0.0001). AUC represents area under curve. Error bars represent mean ± SD.

This data clearly indicate that mutations introduced in S14_M3 and S14_M4 are more detrimental to aptamer binding compared to other mutants (Figure 6C).

DISCUSSION

Currently available antigen detection tests are antibody-based and have moderate sensitivity and specificity. A good antigen test is needed as it provides direct evidence of the presence of the pathogen.[35] Because spike trimeric protein is present on the viral surface and plays a critical role in host cell entry, it is considered an attractive biomarker to diagnose SARS-CoV-2 infection.[36] More recently, an attempt has been made to develop aptamers against the surface protein of other respiratory viruses like influenza and respiratory syncytial virus (RSV), but their clinical diagnostic potential was not demonstrated.[37,38] To the best of our knowledge, there is no report on the application of aptamer as a diagnostic reagent to detect SARS-CoV-2 spike trimeric antigen in clinical specimens (NP swabs). More recently, Song et al.[24] have reported the spike RBD binding aptamers, but their diagnostic utility was not demonstrated. Further, in comparison to RBD region, the complete spike trimer is a large protein that provides ample aptatopes (epitopes) and may allow better binding of aptamers. Thus, in the current investigation, we have used the spike trimeric protein as a target for aptamer generation. As expected, the talon bead-based SELEX approach evince high binding aptamer population enriched against the antigen, the specificity of the enriched population was delineated by comparing the binding with a similar protein (MERS spike). On cloning and sequencing, the R6 aptamer pool resulted in the identification of a panel of aptamers that evinced

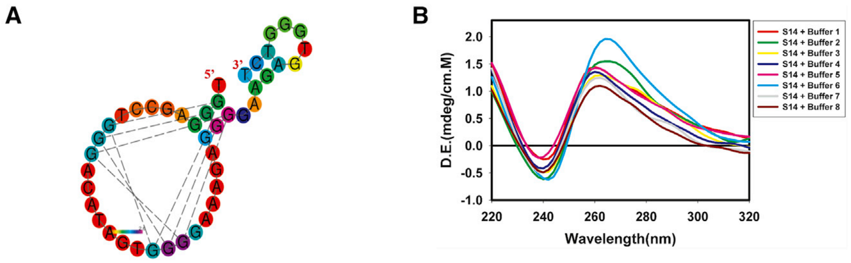

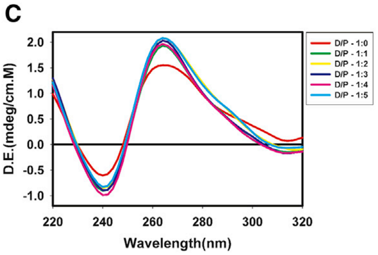

D

aptamer	QGRS sequence	G-Score
S14	GGGACATAGTGGGGAAAGAGGGGAAGA GTGGG	71

Figure 4. Secondary structure prediction of S14 aptamer

(A) RNAfold server by enabling DNA and G-quadruplex parameters to predict the secondary structure of S14 aptamer. (B) CD spectrum for S14 aptamer in different buffers namely buffer 1 (red), buffer 2 (green), buffer 3 (yellow), buffer 4 (blue), buffer 5 (pink), buffer 6 (cyan), buffer 7 (gray), and buffer 8 (brown). (C) CD titration spectrum for S14 aptamer and increasing concentration of spike trimer protein up to the ratio of DNA aptamer (D)/protein (P) = 1:5. (D) The G-quadruplex motif in the S14 aptamer sequence and its G-score predicted using QGRS Mapper. The blue underline guanines participated in the G-quadruplex formation.

high binding to spike protein. Interestingly, post-SELEX optimization yielded two truncated aptamers (S13 and S14) that demonstrated excellent binding and selectivity for spike trimer protein without any cross-reactivity with spike protein of MERS coronavirus, HA protein of influenza virus, HIV-E, and HSA. Surprisingly, upon truncation of aptamer, the binding of aptamer S1 was compromised while the binding of S13 and S14 aptamers remain unaltered. This observation clearly indicates the role of PBD in S1 aptamer binding while truncation of PBD did not affect the binding of S13 and S14 aptamers. These findings are in concordance with the previously reported studies, which highlight the cooperative contribution of aptamer binding to its target.[19,39] Analytical sensitivity of diagnostic reagent is an important parameter that is usually defined by the lowest possible concentration of antigen detected by a molecular recognition element. The S1, S13, and S14 aptamers developed in the current study displayed an impressive limit of detection (LOD, 2 nM) with a linear dynamic range of 0.5–125 nM. The LOD of these aptamers are comparable to that of other antigen-binding aptamers reported in the literature.[23,40,41]

However, when these three aptamers were tested for their clinical diagnostic potential, the discriminatory response of aptamer S13 was inferior in comparison to the other two (S1 and S14) aptamers. This is possibly because of the matrix effect.[42,43] When affinity of S1 and S14 for spike trimer protein was compared, S14 aptamer was found to be superior over S1. High selectivity and affinity of S14 aptamer against the spike trimeric protein can be attributed to the presence of a strong G-quadruplex motifs (G score = 71) in comparison to the S1 and S13 aptamers. CD spectra analysis further corroborated the formation of parallel G-quadruplex topology in the S14 aptamer. In addition to this, CD spectra also reveal the role of cations in the stability of S14 aptamer secondary structure. *In silico* modeling analysis revealed a long single-stranded tail at the 5′ end followed by the formation of a parallel G-quadruplex motif at the 3′ end. To strengthen these findings, G-quadruplex-forming region of S14 aptamer was disrupted that resulted in the compromised binding with the antigen. Molecular docking revealed the binding of S14 with the S1 domain of the spike trimeric protein, where the aptamer interacted with the four amino acids in NTD of the C chain of spike trimeric protein. Thus, *in silico* analysis revealed the preferential binding of S14 with the NTD and was in accordance with the ALISA results, where no binding was observed with the RBD domain of spike protein (Figure 2; Figure S7).

Finally, the performance of the lead aptamer candidate (S14) was assessed in NP specimens obtained from COVID-19-suspected subjects. Importantly, the aptamer-based ALISA demonstrated very high sensitivity and specificity (∼91% sensitivity with ∼98% specificity). This finding indicates comparable efficiency to that of RT-PCR based assay. Further, the performance of ALISA developed in the current study was also compared with other reported antigen and nucleic acid-based diagnostic assays (Table S6). Besides the reported diagnostic assays, the performance of the S14 aptamer in terms of specificity and affinity was found comparable with aptamers developed for viral neutralization.[44,45] The importance of S14 aptamer generated in the current study is underlined by the fact that S14 has not only shown high selectivity and affinity for spike trimer in buffer conditions but also performed equally well in the patient-derived clinical samples (NP swabs), while no previous study has shown the clinical evaluation of aptamers. Taken together, the current study strongly indicates the clinical utility of the ALISA for the diagnosis of SARS-CoV-2 infection. Another important finding of this study is that the spike trimeric binding aptamer, being a synthetic molecule, has not evinced any batch-to-batch variation, which is a vital attribute for a diagnostic reagent. In addition to this, the developed assay can deliver sample-to-answer within ∼5 h with an ability of high throughput screening. Therefore, this assay can be considered a POC test.[46] Unlike molecule diagnostic test that requires extraction and amplification of nucleic acids that demands high-end equipment and infrastructure while ALISA can be operated at routine diagnostic laboratories and health care centers. Interestingly, ALISA developed in this study is compatible with the currently used sample collection strategy that does not

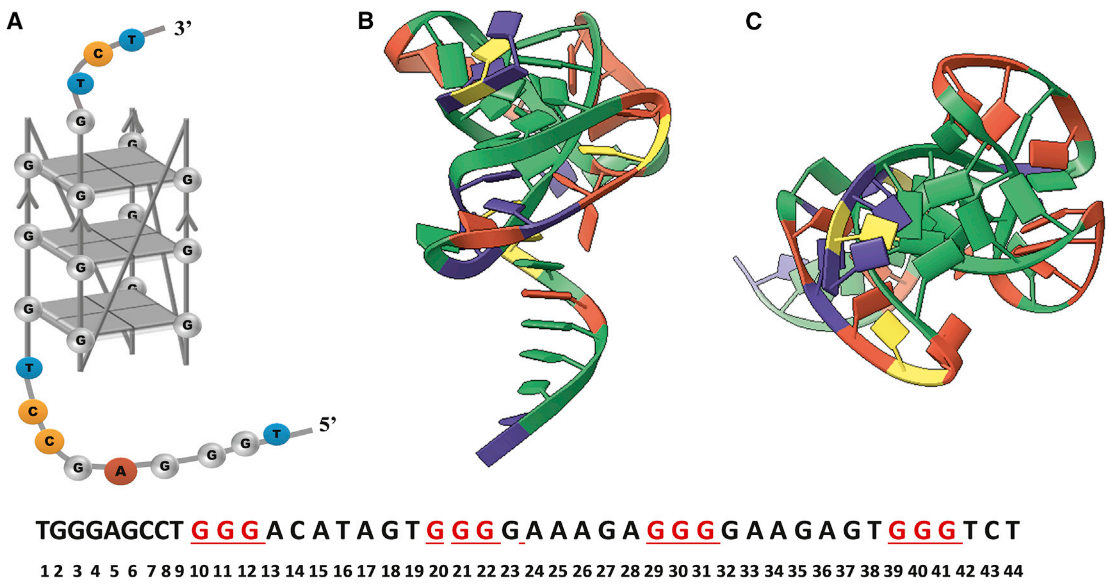

Figure 5. Molecular structure of S14 aptamer constructed based on the best score G-quadruplex motif with the extended chains at 5′ and 3′ ends
(A) The pictorial representation of the aptamer. (B) Optimized tertiary structure of S14. (C) Top view of S14 depicting the stacking of the G-quartets.

require a specialized VTM while commercially available antibody-based antigen detection tests only work with their customized VTM.[4,47] These important qualities may allow the rapid scale-up of a diagnostic test along with consistency in the performance. With the aforementioned advantages associated with aptamers, it has high translational value to be used as an accurate diagnostic test for SARS-CoV-2 to extend our testing capabilities.

Conclusions

Development of an effective vaccine or a drug candidate is a lengthy process as it involves the identification of vaccine/drug candidate, optimization of the same, and validation of the potential vaccine/drug candidate in the animal model followed by clinical trials.[12] In the absence of a vaccine or drug molecule, the number of cases of SARS-CoV-2 may increase day by day across the globe. Considering this situation, the best possible option available to manage this disease is to curtail the viral transmission. This requires rapid and accurate identification of infected persons in a community by employing a robust diagnostic assay. Current diagnostic tests have one or the other limitations such as high turnaround time, low sensitivity or specificity, batch-to-batch variation, dependence on highly skilled manpower, and expensive molecular probes and other reagents. To extend our testing capabilities for SARS-CoV-2, we need a method that could detect the presence of the viral pathogens accurately and can be scaled up rapidly. In the current study, we have developed a high-affinity G-quadruplex-forming DNA aptamer for the spike trimeric protein of SARS-CoV-2. The diagnostic utility of the developed aptamer was demonstrated in the NP specimens. The aptamer-based assay indicates its high translational value for the detection of SARS-CoV-2. At an estimated cost of ∼$2–3 USD/test, we believe this test will supplement our ongoing efforts to tackle the COVID-19 pandemic.

MATERIALS AND METHODS

Reagents and Chemicals

All routine reagents were procured from Sigma Aldrich, USA, unless otherwise mentioned. Oligonucleotides used in the study were procured from Integrated DNA Technologies (IDT, USA), Bioserve Biotechnologies (India), and Macrogen (South Korea). 96-well plates (MaxiSorp) were procured from Nunc, USA. Polyvinylidene fluoride (PVDF) membrane was obtained from Merck, USA. Bovine serum albumin (BSA) and nuclease free water (NFW) were procured from Himedia Laboratories, India. 3, 3′, 5, 5′-tetramethylbenzidine (TMB; BD OptEIA) was procured from BD Biosciences, USA. PCR master mix obtained from EmeraldAmp PCR master mix TakaraBio, Japan. Following antibodies are used in this study: anti-His antibody (Thermo Fisher Scientific, USA), anti-HIV-1 gp120 Env (ABLinc, USA), anti-HSA monoclonal antibody (R&D Systems, USA).

Expression and purification of SARS-CoV-2 prefusion-stabilized spike trimer

Prefusion stabilized ectodomain construct of SARS-CoV-2 spike trimer was a gift from Barney S. Graham's lab (NIH).[48] The ectodomain construct with 2P mutation, C-terminal T4 fibritin trimerization motif to stabilized trimeric conformation, and Twin StrepTag and 8X-HisTag, was expressed in fully glycosylated form through mammalian expression system (Expi 293 cells, Thermo Fisher, USA) following the manufacturer's protocol. Briefly, Expi293 cells were transfected with plasmid DNA using ExpiFectamine 293 Transfection Kit (Thermo Fisher, USA). The transfected supernatant with secreted protein was harvested 6 h post-transfection by centrifugation of the transfected culture. The harvested supernatant with spike trimer was loaded onto Ni-NTA agarose (QIAGEN, USA), equilibrated with 50 mM Tris-HCl pH 7.4 and

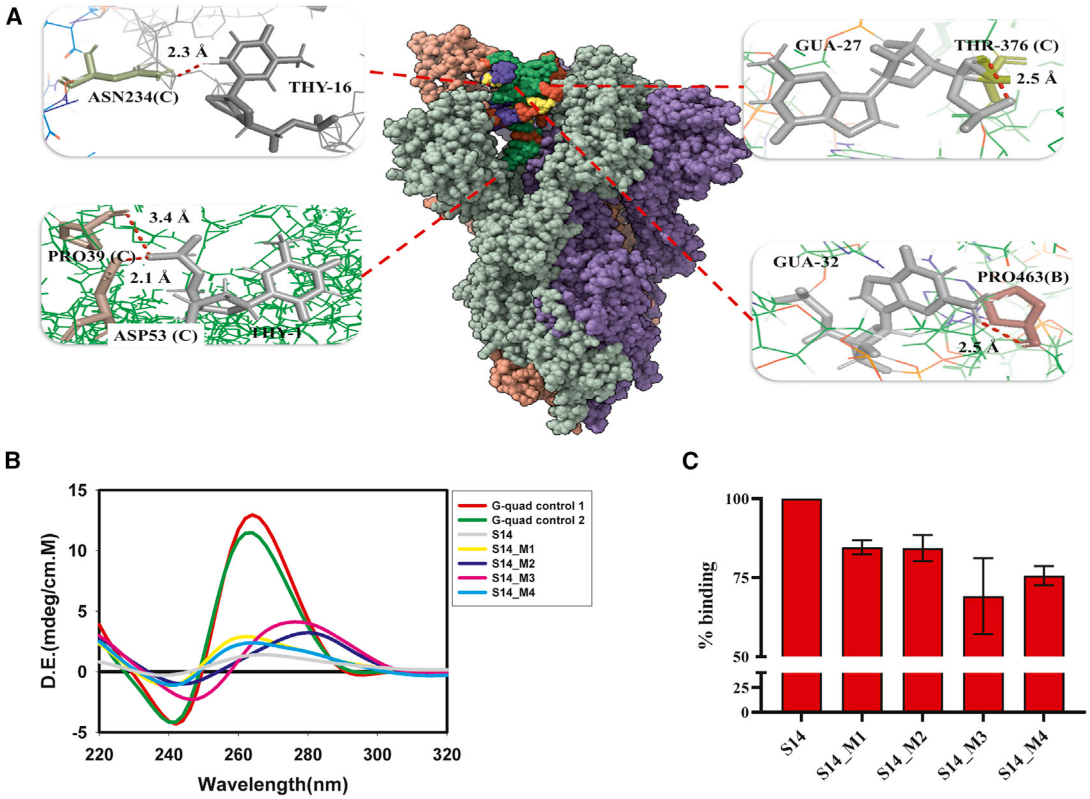

Figure 6. Molecular interaction and mutational analysis for S14 aptamer

(A) S14 aptamer with the FL spike trimer protein (surface representation). A, B, and C chain of spike protein is colored blue, raspberry, and sea green, respectively, while aptamer is colored on the basis of its nucleotide composition using ChimeraX 1.0. The interacting residues are represented by stick representation with the H-bonds in red color. (B) CD spectrum for S14 mutants namely S14_M1 (yellow), S14_M2 (blue), S14_M3 (pink), and S14_M4 (cyan) with two control aptamers (red and green) and parent S14 (gray). (C) Impact of mutations on aptamer binding to spike antigen. Binding of each mutant was compared against parent aptamer S14. In this experiment binding of S14 aptamer to spike antigen was considered as 100%. Error bars represent mean ± SD.

100 mM NaCl at 4°C temperature utilizing gravitational flow, the column was washed further with equilibration buffer, and protein was eluted with elution buffer containing 500 mM Imidazole. The purity of eluted fractions was assessed on SDS-PAGE and western blotting using "SARS/SARS-CoV-2 Coronavirus Spike Protein (subunit 1) Polyclonal Antibody" (Thermo Fisher Scientific, USA). Ni-NTA purified fractions were then pooled and dialyzed against 1× PBS. The dialyzed spike trimeric protein was further subdivided into small aliquots, snap-frozen in liquid nitrogen, and stored at −80°C until further use.

Aptamer development through SELEX

A Talon bead-based SELEX strategy was followed for aptamer selection as described previously with few modifications.[49] A mixture of aptamer libraries were used that had a central core of 41–44 nucleotides (N41–N44) and flanked by 18-mer PBD on either side to enable DNA amplification by PCR using DRF as forward primer (forward, 5′-GTCTTGACTAGTTACGCC-3′) and DRR as reverse primer (reverse, 5′-GAGGCGCCAACTGAATGA-3′).[31] From the PCR amplicon ssDNA was prepared using ribo-linkage containing reverse primer followed by alkaline lysis and Urea-PAGE as described previously.[31] To select aptamers against the spike trimer, aptamer library was annealed and used for negative selection with talon-beads followed by positive selection using His_8-tagged spike trimer (10 μg) immobilized on talon beads in BB (1× PBS pH 7.4, 10 mM $MgCl_2$, 50 mM KCl, 25 mM NaCl). The spike binding aptamer pool was eluted and enriched as described previously.[31] This enriched pool of aptamer was made single stranded and used for next round of SELEX. After six rounds of selection, the aptamer pool was cloned in pTZ57R/T vector system (Thermo Fisher Scientific InsTAclone PCR cloning kit) and transformed in *E. coli* DH5α. The obtained colonies were randomly picked and analyzed by DNA sequencing.

ALISA

In brief, 125 ng/well spike trimer protein was coated in 96-well plates at 37°C for 2 h in 1× PBS. Wells were blocked with 1% BSA in BB for 1 h at RT. After blocking, wells were washed with BB once, and then 50 picomoles of biotin labeled aptamers were added to each well for 1 h at RT. The wells were washed four times with wash buffer (WB; BB supplemented with 0.5% T-20). Streptavidin-horseradish peroxidase (HRP) was added at 1:3,000 dilutions with 0.05% T-20 to each

well and incubated for 1 h at RT. Post incubation; wells were washed with WB and were developed using 100 μL TMB substrate. The reaction was quenched using 5% H_2SO_4, and optical density (OD) was measured at 450 nm on ELISA reader. ΔOD 450 nm estimated by subtracting OD 450 nm of appropriate negative controls (aptamer control, antigen control, etc.) was plotted.

Evaluation of cross-reactivity of DNA aptamers

To assess the cross-reactivity of SELEX-derived aptamers with other antigens, we selected the three best performing aptamer candidates. The cross-reactivity of these three aptamer candidates was assessed with various antigens, namely, RBD of SARS-CoV-2, MERS spike, HIV-E monomer, HIV-E trimer, Influenza HA monomer, Influenza HA trimer, and HSA in an ALISA as described in the previous section.

Analytical sensitivity of aptamers

The analytical sensitivity of the three best performing aptamer candidates was determined in terms of the lowest concentration of antigen detected by these aptamers. Briefly, a range (2–500 nM/well) of spike trimer protein was coated on 96-well plates and binding of each aptamer candidate was assessed across the aforementioned range of protein concentration in an ALISA. Finally, OD450 nm was plotted as a function of spike trimer concentration to determine the low-end detection limit of each aptamer candidate.

Clinical diagnostic application of aptamers

NP swabs (n = 232) collected at clinical sites for COVID-19 investigation were subjected to RT-PCR at the bioassay laboratory, THSTI. NP swabs were parallelly tested using aptamer-based assay (ALISA) in a blinded manner. The sample was decoded after the completion of ALISA. Next to this, ALISA data were compared with RT-PCR data. Briefly, NP swab samples were diluted 1:25 (12 μL VTM + 288 μL 1× PBS) at final volume of 300 μL and incubated for 2 h at 37°C. Post incubation, wells were washed with BB, and the remaining procedure for ALISA was same as described above. All participants were enrolled after a duly informed consent process and Institute Ethics Committees of the participating research institutes and hospitals (DBT research consortium) approved, THS 1.8.1/ (91) the protocols for this study.

BLI

BLI experiments were done using Octet Red96 instrument (ForteBio, USA). A 25 nM concentration of aptamer S1 and S14 were immobilized on a streptavidin-coated biosensor surface. The reference sensors were blocked using biotinylated BSA (50 μg/mL) to reduce non-specific background binding. The baseline interference phase was obtained by measurements taken for 60 s in BB supplemented with 0.02% Tween-20. Next to this, the sensors were subjected to association phase immersion for 300 sec in wells containing different concentrations of spike trimer diluted in BB. Following the association phase, the sensors were immersed into BB for an additional 300 sec to estimate the dissociation. The K_{on}, and apparent K_D values of the aptamers S1 and S14 were estimated to establish their binding affinities for spike trimer. K_D value was determined utilizing 1:1 global fit model. The fittings were considered to be satisfactory if $\chi 2 < 0.5$. Graphs were plotted using Origin v8.0.

CD spectral analysis

To analyze the secondary structure of the aptamers, we performed CD using Jasco J-1500 spectrophotometer (Jasco Hachioji, Tokyo, Japan) equipped with a Peltier temperature controller.[50] A continuous supply of nitrogen gas was purged to avert water condensation around the cuvette, if any. The aptamer samples were dissolved in the BB with a working concentration of 20 μM and spectra was analyzed in a 2 mm cuvette in a range of 200–340 nm. Scanning speed was fixed at 20 nm/min. To nullify the BB effect on the spectra, we obtained a blank spectrum for the BB and subtracted it from the spectra of the aptamer. In addition to this, to assess the effect of salt, various buffers (pH 7.4) were prepared in different combinations of salt viz buffer 1 (1× PBS only), buffer 2 (1× PBS + 10 mM $MgCl_2$ + 25 mM NaCl + 50 mM KCl), buffer 3 (1× PBS + 10 mM $MgCl_2$), buffer 4 (1× PBS + 50 mM KCl), buffer 5 (1× PBS + 25 mM NaCl), buffer 6 (1× PBS + 10 mM $MgCl_2$ + 50 mM KCl), buffer 7 (1× PBS + 25 mM NaCl + 50 mM KCl), and buffer 8 (1× PBS + 10 mM $MgCl_2$ + 25 mM NaCl) for CD spectra of S14 in these buffers. Also, CD spectra was obtained to observe the target-dependent changes occur in the structure of aptamer in response to increasing concentration of spike antigen. CD graphs were plotted using Sigma Plot v8.5.

Structure prediction of S14 and its molecular docking with the spike trimer

The best performing aptamer candidate (S14) was analyzed for its G-quadruplex-forming propensity using QGRS Mapper.[51] The parameters used for the prediction was a minimum G-tract of 3Gs with a loop length of 7 and a maximum length of 45 bases. The G4 motif with the highest G-score was selected for its structure prediction using the 3D-Nus server.[52] The extra 5′ and 3′ nucleotide bases were added using Discovery Studio v4.0 (DS).[53] The resultant structure was energy minimized by exploiting the conjugate gradient algorithm in DS. Molecular docking of the S14 aptamer with spike trimer protein (PDB ID: 6VYB) was performed using standalone Hex 8.0.0 software on a Linux environment. The docked complex was analyzed by using ChimeraX 1.0 and open source Pymol.

S14 aptamer mutant's analysis

Structure guided four structural variants of S14 (S14_M1, S14_M2, S14_M3, and S14_M4) were generated by disrupting the G-quadruplex forming region. Binding of each of these mutants were compared with parent aptamer S14 by ALISA as described in the previous section.

Statistical analysis

All experimental data are represented as mean ± SD determined using GraphPad Prism v7.0 software. To evaluate the diagnostic potential of SARS-CoV-2 spike trimer in ALISA, we used OD_{450} values obtained in NP swabs from suspected COVID-19 patients to construct a ROC curve using GraphPad Prism. The cut-off points were derived to achieve ≥98% test specificity. The test sensitivity was calculated on this cut-off point using real-time PCR as a gold standard.

SUPPLEMENTAL INFORMATION

Supplemental information can be found online at https://doi.org/10.1016/j.omtn.2021.06.014.

CONSORTIA

The members of the DBT India Consortium for Covid-19 Research are Shinjini Bhatnagar, Gagandeep Kang, Nitya Wadhwa, Uma Chandramouli Natchu, Ramachandran Thiruvengadam, Shailaja Sopory, Pallavi Kshetrapal, Bapu Koundinya Desiraju, Vandita Bhartia, Mudita Gosain, Gaurav Batra, Guruprasad Medigeshi, Susmita Chaudhuri, Niraj Kumar, Tarun Sharma, Chandresh Sharma, Shailendra Mani, Tripti Shrivastava, Monika Bahl, Anmol Chandele, Vijay Kumar Chaudhary, Amulya Panda, Nimesh Gupta, Nandini Sharma, Pragya Sharma, Sonal Saxena, J.C. Passey, Suresh Kumar, Anil K Pandey, Asim Das, Nikhil Verma, Needhi Anand, Sujata Roy Choudhary, Deepa Sindhu, Jai Singh Malik, Brahmdeep Sindhu, Bhupinder Kaur Anand, Shubham Girdhar, Sushila Kataria, Pooja Sharma, Dr Yamini, Harish K. Pemde, Tanmaya Talukdar, Pankaj Abrol, Mukesh Sharma, Navin Dang, Manavi Dang, Arjun Dang, Leena Chatterjee, and Devjani De.

ACKNOWLEDGMENTS

T.K.S. thanks the Translational Health Science & Technology Institute, Faridabad for providing core grant support, IndCEPI Mission (BT/MB/CEPI/2016) and Translational Research Program (BT/PR30159/MED/15/188/2018). A.G. and A. Anand are thankful to CEPI (Coalition for Epidemic Preparedness Innovations) for providing the financial assistantship. HIV protein for cross-reactivity assessment was provided as a kind gift from Dr. Rajesh Kalra, THSTI, Faridabad. We thank Dr. Jae-Ouk Kim, International Vaccine Institute (IVI), Gwanak-gu, Seoul, South Korea for generously providing the MERS-spike protein. We would like to thank Bioassay lab, THSTI, for providing access to perform all clinical assays. The excellent technical assistance provided by Mr. Raj Kumar Dwivedi is duly acknowledged. We also thank Advance Technology Platform Center (ATPC), NCR biotech science cluster, Faridabad, for sequencing and BLI facility.

AUTHOR CONTRIBUTIONS

T.K.S., A.K., G.R.M., T.S., and S.B. designed and supervised the experiments. A.G., A. Anand, N.J., S.G., A. Ananthraj, S.P., and R.S. performed experiments. A.G., A. Anand, N.J., S.G., A. Ananthraj, S.P., R.S., T.K.S., A.K., G.R.M., and T.S. collected and analyzed the data. T.K.S., A.K., T.S., A.G., A. Anand, and N.J. wrote the manuscript. A.G. and A. Anand contributed equally in conducting experiments, acquiring data, analyzing data, and writing the manuscript. All authors reviewed the manuscript.

DECLARATION OF INTERESTS

The aptamer sequences reported in this study are proprietary reagents and deposited in the Indian Patent Office as a part of Indian Patent application no. 202011017852. This technology has transferred to Mol-Bio Diagnostics Pvt. Ltd., India for scale-up and commercialization.

REFERENCES

1. El Zowalaty, M.E., and Järhult, J.D. (2020). From SARS to COVID-19: A previously unknown SARS- related coronavirus (SARS-CoV-2) of pandemic potential infecting humans - Call for a One Health approach. One Health *9*, 100124.
2. Yamin, M. (2020). Counting the cost of COVID-19. Int J Inf Technol *12*, 1–7.
3. Abdelrahman, Z., Li, M., and Wang, X. (2020). Comparative Review of SARS-CoV-2, SARS-CoV, MERS-CoV, and Influenza A Respiratory Viruses. Front Immunol. *11*, 552909.
4. Feng, W., Newbigging, A.M., Le, C., Pang, B., Peng, H., Cao, Y., Wu, J., Abbas, G., Song, J., Wang, D.B., et al. (2020). Molecular Diagnosis of COVID-19. Anal. Chem. *92*, 10196–10209.
5. Tay, M.Z., Poh, C.M., Rénia, L., MacAry, P.A., and Ng, L.F.P. (2020). The trinity of COVID-19: immunity, inflammation and intervention. Nat. Rev. Immunol. *20*, 363–374.
6. World Health Organization (2021). Coronavirus Disease (COVID-19) Situation Reports, https://www.who.int/emergencies/diseases/novel-coronavirus-2019/situation-reports.
7. Badgujar, K.C., Badgujar, V.C., and Badgujar, S.B. (2020). Vaccine development against coronavirus (2003 to present): An overview, recent advances, current scenario, opportunities and challenges. Diabetes Metab Syndr. *14*, 1361–1376.
8. Wouters, O.J., Shadlen, K.C., Salcher-Konrad, M., Pollard, A.J., Larson, H.J., Teerawattananon, Y., and Jit, M. (2021). Challenges in ensuring global access to COVID-19 vaccines: production, affordability, allocation, and deployment. Lancet *397*, 1023–1034.
9. Dal-ré, R., Bekker, L., Gluud, C., Holm, S., Jha, V., Poland, G.A., Rosendaal, F.R., Schwarzer-daum, B., Sevene, E., Tinto, H., et al. (2021). Personal View Ongoing and future COVID-19 vaccine clinical trials: challenges and opportunities. Lancet Infect Dis. *3099*, 19–24.
10. Carter, L.J., Garner, L.V., Smoot, J.W., Li, Y., Zhou, Q., Saveson, C.J., Sasso, J.M., Gregg, A.C., Soares, D.J., Beskid, T.R., et al. (2020). Assay Techniques and Test Development for COVID-19 Diagnosis. ACS Cent. Sci. *6*, 591–605.
11. Leenaars, M., and Hendriksen, C.F.M. (2005). Critical steps in the production of polyclonal and monoclonal antibodies: evaluation and recommendations. ILAR J. *46*, 269–279.
12. Berglund, L., Björling, E., Oksvold, P., Fagerberg, L., Asplund, A., Szigyarto, C.A.K., Persson, A., Ottosson, J., Wernérus, H., Nilsson, P., et al. (2008). A genecentric Human Protein Atlas for expression profiles based on antibodies. Mol. Cell. Proteomics *7*, 2019–2027.
13. Bauer, M., Strom, M., Hammond, D.S., and Shigdar, S. (2019). Anything you can do, i can do better: Can aptamers replace antibodies in clinical diagnostic applications? Molecules *24*, 1–13.
14. Baker, M. (2015). Reproducibility crisis: Blame it on the antibodies. Nature *521*, 274–276.
15. Hou, H., Wang, T., Zhang, B., Luo, Y., Mao, L., Wang, F., Wu, S., and Sun, Z. (2020). Detection of IgM and IgG antibodies in patients with coronavirus disease 2019. Clin. Transl. Immunology *9*, 1–8.
16. Dhar, P., Samarasinghe, R.M., and Shigdar, S. (2020). Antibodies, Nanobodies, or Aptamers — Which Is Best for Deciphering the Proteomes of Non-Model Species? Int. J. Mol. Sci. *21*, 2485.
17. Kaur, H., Bruno, J.G., Kumar, A., and Sharma, T.K. (2018). Aptamers in the therapeutics and diagnostics pipelines. Theranostics *8*, 4016–4032.
18. Acquah, C., Danquah, M.K., Agyei, D., Moy, C.K.S., Sidhu, A., and Ongkudon, C.M. (2016). Deploying aptameric sensing technology for rapid pandemic monitoring. Crit. Rev. Biotechnol. *36*, 1010–1022.
19. Sharma, T.K., Bruno, J.G., and Dhiman, A. (2017). ABCs of DNA aptamer and related assay development. Biotechnol. Adv. *35*, 275–301.
20. Romero-López, C., and Berzal-Herranz, A. (2017). Aptamers: Biomedical interest and applications. Pharmaceuticals (Basel) *10*, 32.
21. Rossi, J.J., and Rossi, D. (2020). Oligonucleotides and the COVID-19 Pandemic: A Perspective. Nucleic Acid Ther. *30*, 129–132.

22. Dhiman, A., Kumar, C., Mishra, S.K., Sikri, K., Datta, I., Sharma, P., Singh, T.P., Haldar, S., Sharma, N., Bansal, A., et al. (2019). Theranostic Application of a Novel G-Quadruplex-Forming DNA Aptamer Targeting Malate Synthase of Mycobacterium tuberculosis. Mol. Ther. Nucleic Acids *18*, 661–672.

23. Dhiman, A., Anand, A., Malhotra, A., Khan, E., Santra, V., Kumar, A., and Sharma, T.K. (2018). Rational truncation of aptamer for cross-species application to detect krait envenomation. Sci. Rep. *8*, 17795.

24. Song, Y., Song, J., Wei, X., Huang, M., Sun, M., Zhu, L., Lin, B., Shen, H., Zhu, Z., and Yang, C. (2020). Discovery of Aptamers Targeting the Receptor-Binding Domain of the SARS-CoV-2 Spike Glycoprotein. Anal. Chem. *92*, 9895–9900.

25. Tang, X.L., Zhou, Y.X., Wu, S.M., Pan, Q., Xia, B., and Zhang, X.L. (2014). CFP10 and ESAT6 aptamers as effective Mycobacterial antigen diagnostic reagents. J. Infect. *69*, 569–580.

26. Ray, P., Rialon-Guevara, K.L., Veras, E., Sullenger, B.A., and White, R.R. (2012). Comparing human pancreatic cell secretomes by in vitro aptamer selection identifies cyclophilin B as a candidate pancreatic cancer biomarker. J. Clin. Invest. *122*, 1734–1741.

27. Zeng, Z., Zhang, P., Zhao, N., Sheehan, A.M., Tung, C.H., Chang, C.C., and Zu, Y. (2010). Using oligonucleotide aptamer probes for immunostaining of formalin-fixed and paraffin-embedded tissues. Mod. Pathol. *23*, 1553–1558.

28. Lavania, S., Das, R., Dhiman, A., Myneedu, V.P., Singh, N., Sharma, T.K., and Tyagi, J.S. (2018). Aptamer-based TB antigen tests for the rapid diagnosis of pulmonary tuberculosis: Potential utility in screening for tuberculosis. ACS Infect Dis. *4*, 1718–1726.

29. Kumari, P., Lavania, S., Tyagi, S., Dhiman, A., Rath, D., Anthwal, D., Gupta, R.K., Sharma, N., Gadpayle, A.K., Taneja, R.S., et al. (2019). A novel aptamer-based test for the rapid and accurate diagnosis of pleural tuberculosis. Anal. Biochem. *564-565*, 80–87.

30. Gruber, A.R., Lorenz, R., Bernhart, S.H., Neuböck, R., and Hofacker, I.L. (2008). The Vienna RNA websuite. Nucleic Acids Res. *36*, W70-4.

31. Kalra, P., Mishra, S.K., Kaur, S., Kumar, A., Prasad, H.K., Sharma, T.K., and Tyagi, J.S. (2018). G-Quadruplex-Forming DNA Aptamers Inhibit the DNA-Binding Function of HupB and Mycobacterium tuberculosis Entry into Host Cells. Mol. Ther. Nucleic Acids *13*, 99–109.

32. Jain, N., Mishra, S.K., Shankar, U., Jaiswal, A., Sharma, T.K., Kodgire, P., et al. (2020). G-quadruplex stabilization in the ions and maltose transporters gene inhibit Salmonella enterica growth and virulence. Genomics *112*, 4863–4874.

33. Bhattacharyya, D., Mirihana Arachchilage, G., and Basu, S. (2016). Metal cations in G-quadruplex folding and stability. Front Chem. *4*, 38.

34. Owczarzy, R., Moreira, B.G., You, Y., Behlke, M.A., and Wälder, J.A. (2008). Predicting stability of DNA duplexes in solutions containing magnesium and monovalent cations. Biochemistry *47*, 5336–5353.

35. Harapan, H., Itoh, N., Yufika, A., Winardi, W., Keam, S., Te, H., Megawati, D., Hayati, Z., Wagner, A.L., and Mudatsir, M. (2020). Coronavirus disease 2019 (COVID-19): A literature review. J. Infect. Public Health *13*, 667–673.

36. Wang, L., Wang, Y., Ye, D., and Liu, Q. (2020). Review of the 2019 novel coronavirus (SARS-CoV-2) based on current evidence. Int. J. Antimicrob. Agents *55*, 105948.

37. Percze, K., Szakács, Z., Scholz, É., András, J., Szeitner, Z., Kieboom, C.H., Ferwerda, G., Jonge, M.I.D., Gyurcsányi, R.E., and Mészáros, T. (2017). Aptamers for respiratory syncytial virus detection. Sci. Rep. *7*, 42794.

38. Zou, X., Wu, J., Gu, J., Shen, L., and Mao, L. (2019). Application of aptamers in virus detection and antiviral therapy. Front. Microbiol. *10*, 1462.

39. Eaton, B.E., Gold, L., Hicke, B.J., Janjić, N., Jucker, F.M., Sebesta, D.P., Tarasow, T.M., Willis, M.C., and Zichi, D.A. (1997). Post-SELEX combinatorial optimization of aptamers. Bioorg. Med. Chem. *5*, 1087–1096.

40. Giri, B., Pandey, S., Shrestha, R., Pokharel, K., Ligler, F.S., and Neupane, B.B. (2020). Review of analytical performance of COVID-19 detection methods. Anal. Bioanal. Chem. *413*, 35–48.

41. Dhiman, A., Kalra, P., Bansal, V., Bruno, J.G., and Kumar, T. (2017). Sensors and Actuators B : Chemical Aptamer-based point-of-care diagnostic platforms. Sens. Actuators B Chem. *246*, 535–553.

42. Das, R., Sharma, M.K., Rao, V.K., Bhattacharya, B.K., Garg, I., Venkatesh, V., and Upadhyay, S. (2014). An electrochemical genosensor for Salmonella typhi on gold nanoparticles-mercaptosilane modified screen printed electrode. J. Biotechnol. *188*, 9–16.

43. Hong, P., Li, W., and Li, J. (2012). Applications of aptasensors in clinical diagnostics. Sensors (Basel) *12*, 1181–1193.

44. Sun, M., Liu, S., Wei, X., Wan, S., Huang, M., Song, T., Lu, Y., Weng, X., Lin, Z., Chen, H., et al. (2021). Aptamer Blocking Strategy Inhibits SARS-CoV-2 Virus Infection. Angew. Chemie Int. Ed. Engl. *60*, 10266–10272.

45. Schmitz, A., Weber, A., Bayin, M., Breuers, S., Fieberg, V., Famulok, M., and Mayer, G. (2021). A SARS-CoV-2 Spike Binding DNA Aptamer that Inhibits Pseudovirus Infection by an RBD-Independent Mechanism*. Angew. Chem. Int. Ed. Engl. *60*, 10279–10285.

46. Kaur, H., Dhiman, A., Bruno, J.G., and Sharma, T.K. (2019). Defining Target Product Profiles (TPPs) for Aptamer-Based Diagnostics. Advances in Biochemical Engineering/Biotechnology *174*, 195–209.

47. Mahapatra, S., and Chandra, P. (2020). Clinically practiced and commercially viable nanobio engineered analytical methods for COVID-19 diagnosis. Biosens. Bioelectron. *165*, 112361.

48. Wrapp, D., Wang, N., Corbett, K.S., Goldsmith, J.A., Hsieh, C., Abiona, O., Graham, B.S., and Mclellan, J.S. (2020). Cryo-EM structure of the 2019-nCoV spike in the prefusion conformation. Science *367*, 1260–1263.

49. Dhiman, A., Haldar, S., Mishra, S.K., Sharma, N., Bansal, A., Ahmad, Y., Kumar, A., Sharma, T.K., and Tyagi, J.S. (2018). Generation and application of DNA aptamers against HspX for accurate diagnosis of tuberculous meningitis. Tuberculosis (Edinb.) *112*, 27–36.

50. Kypr, J., Kejnovská, I., Renčiuk, D., and Vorlícková, M. (2009). Circular dichroism and conformational polymorphism of DNA. Nucleic Acids Res. *37*, 1713–1725.

51. Kikin, O., D'Antonio, L., and Bagga, P.S. (2006). QGRS Mapper: a web-based server for predicting G-quadruplexes in nucleotide sequences. Nucleic Acids Res. *34*, W676-82.

52. Patro, L.P.P., Kumar, A., Kolimi, N., and Rathinavelan, T. (2017). 3D-NuS: A Web Server for Automated Modeling and Visualization of Non-Canonical 3-Dimensional Nucleic Acid Structures. J. Mol. Biol. *429*, 2438–2448.

53. Šmidlehner, T., Piantanida, I., and Pescitelli, G. (2018). Polarization spectroscopy methods in the determination of interactions of small molecules with nucleic acids - tutorial. Beilstein J. Org. Chem. *14*, 84–105.

OMTN, Volume *26*

Supplemental information

A novel G-quadruplex aptamer-based spike trimeric antigen test for the detection of SARS-CoV-2

Ankit Gupta, Anjali Anand, Neha Jain, Sandeep Goswami, Anbalagan Anantharaj, Sharanabasava Patil, Rahul Singh, Amit Kumar, Tripti Shrivastava, Shinjini Bhatnagar, Guruprasad R. Medigeshi, Tarun Kumar Sharma, and DBT India Consortium for COVID-19 Research

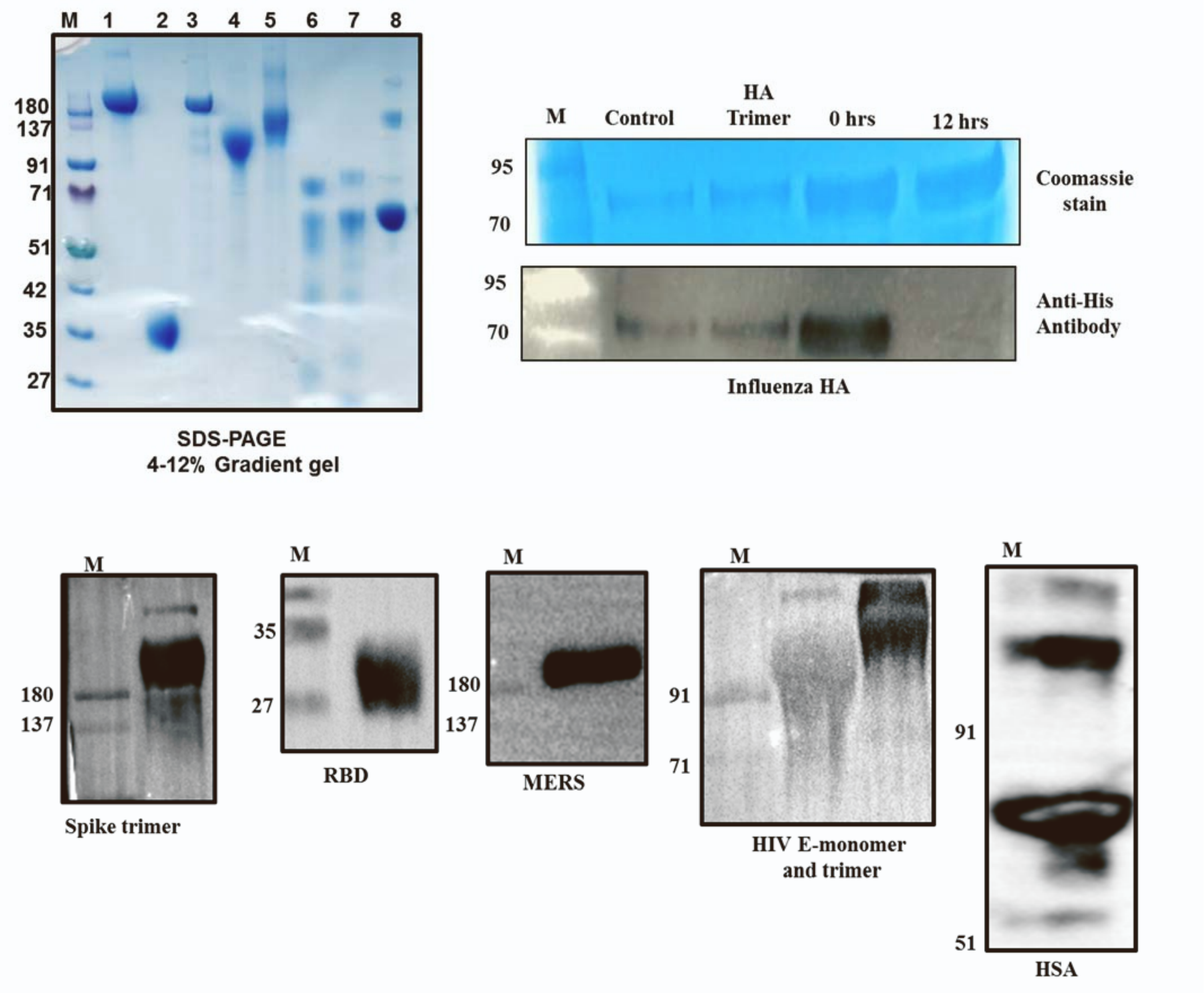

Figure S1. SDS-PAGE electrophoretogram of various proteins used in the study. lane 1 spike trimer protein (~194 kDa), lane 2 SARS spike-RBD (~ 32 kDa), lane 3 MERS FL-spike (~ 190 kDa), lane 4 HIV-E monomer (~ 95kDa), lane 5 HIV-E trimer (~ 130kDa), lane 6 Influenza HA monomer (~ 75 kDa), lane 7 Influenza HA trimer (~ 82 kDa) and lane 8 HSA (~ 66 kDa) on a gradient gel (4-12%). Western blot was developed using either the protein specific or anti-his antibody. M denotes the protein ladder in kDa.

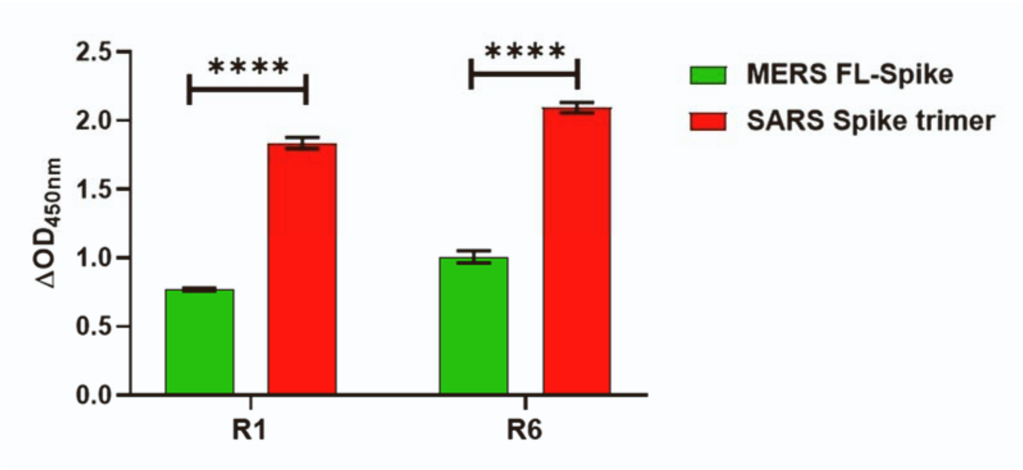

Figure S2. Comparing relative binding of round population (R1 and R6) enriched against spike trimer with MERS full length (FL)-spike. **** represent statistical significance at p value (***p < 0.0001). To compare the binding two-way ANOVA with multiple comparison was applied. Bars represent mean ± SD. ΔOD 450nm represents OD of wells with antigen subtracted OD of wells without antigen.

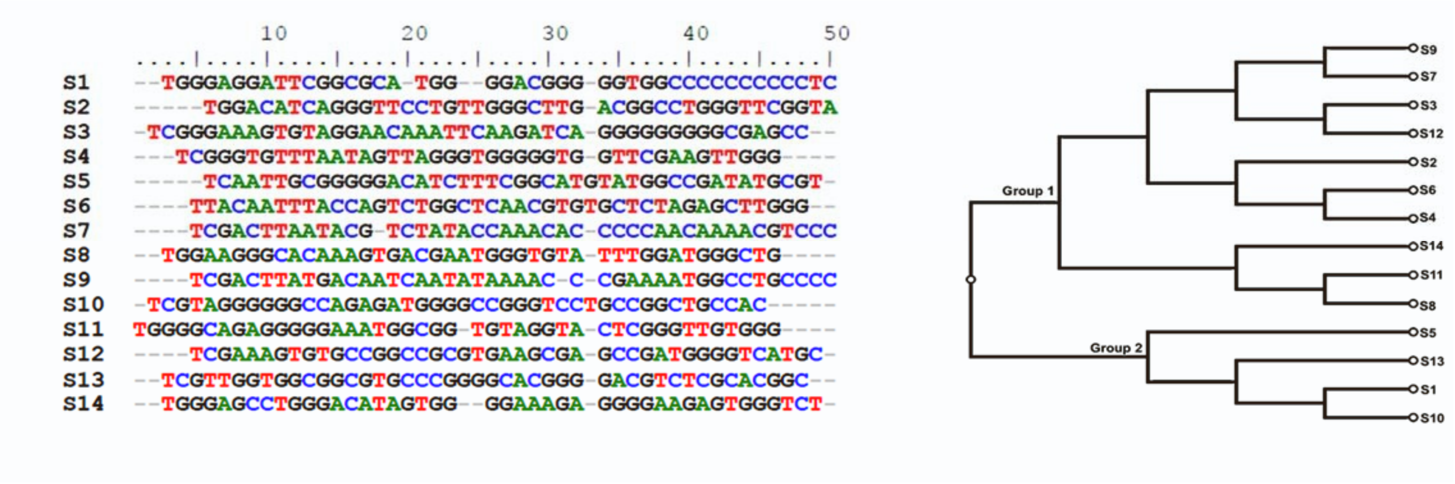

Figure S3. Multiple sequence alignment of 14 aptamer candidates targeting spike trimer antigen and ClustalW generated rooted phylogenetic tree analysis (UPGMA) for the aptamer candidates.

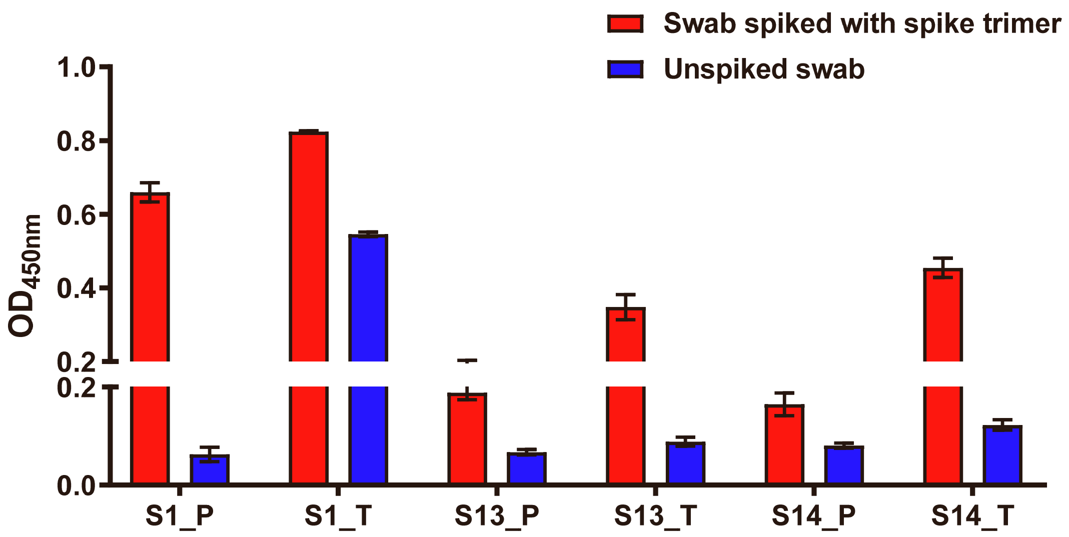

Figure S4. Comparison of binding performance parent (P) versus truncated (T) version of S1, S13 and S14 aptamer in viral transport media (VTM) spiked with antigen using ALISA. Bars represent mean ± SD.

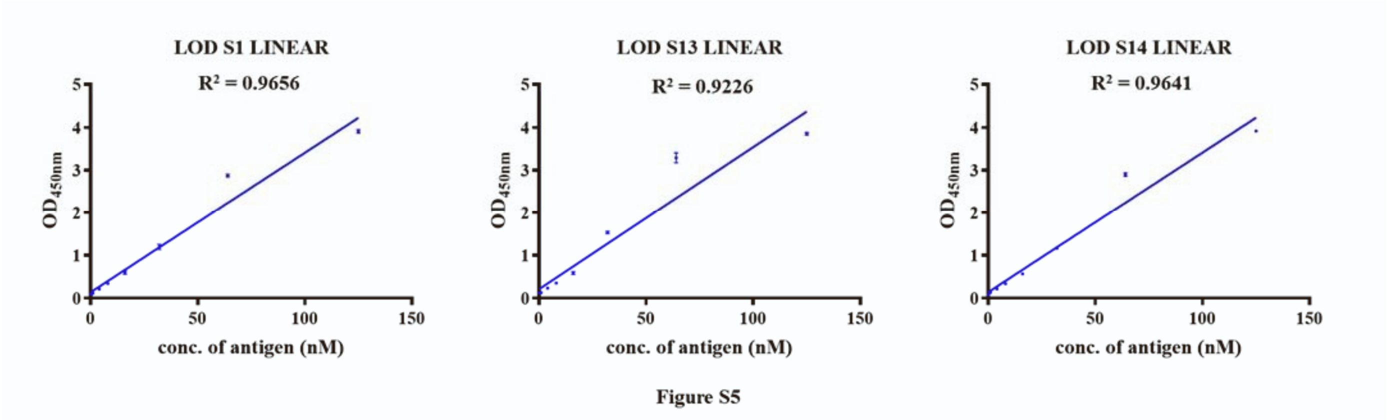

Figure S5. Linear dynamic range for 3 aptamers (S1, S13 and S14) from antigen concentration of 0.5-125 nM with a linearity regression coefficient (r^2) of >0.9226.

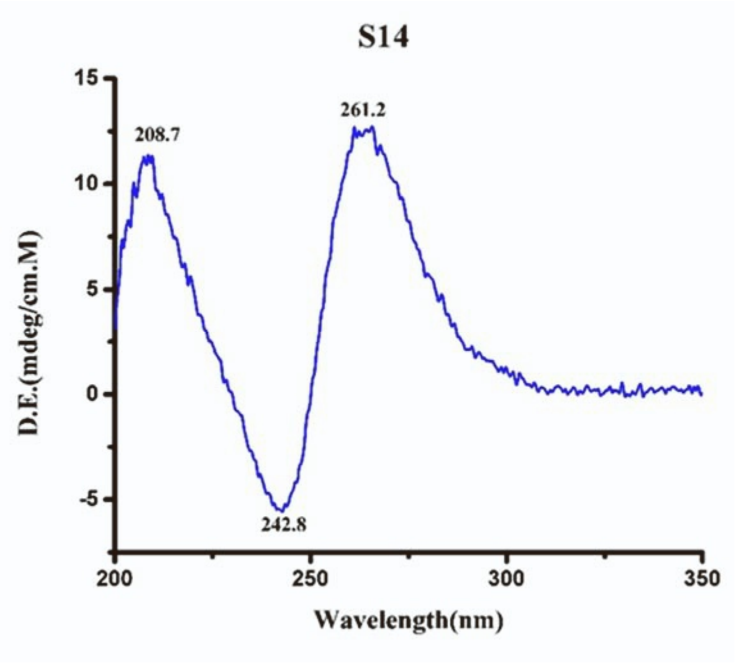

Figure S6. CD spectrum of S14 aptamer showing parallel G-quadruplex structure with signature peak at ~ 242 nm (negative) and ~ 261 nm (positive).

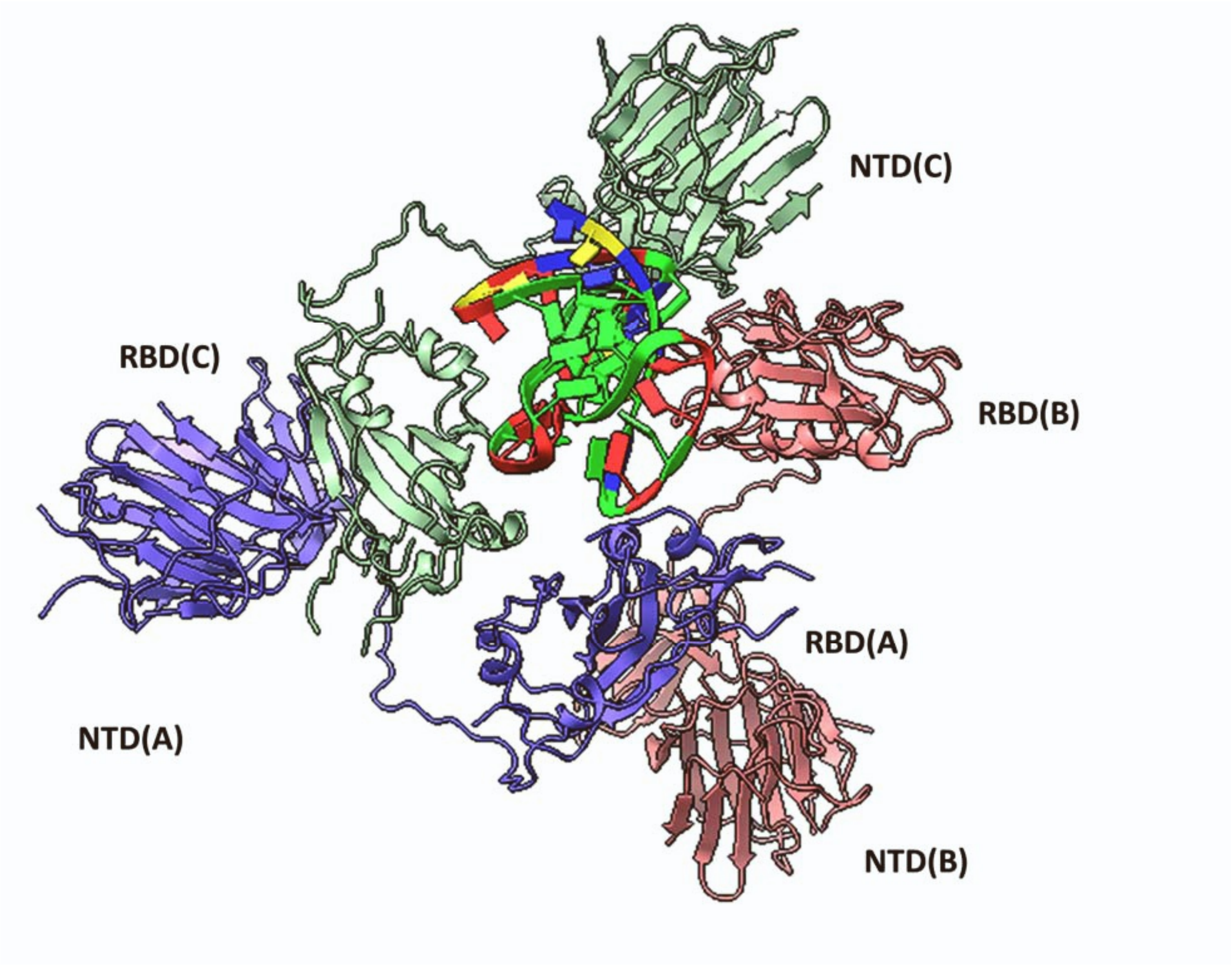

Figure S7. Top view of spike trimeric protein (showing only the NTD and RBD domains of all the three chains) in complex with the S14 aptamer. Aptamer is interacting with NTD(C) and RBD(B).

Table S1. MEME suite predicted motif in the aptamer sequences

Motif No.	Motif	E-value	% Occurence	Start Position in aptamers (nt no.)
1	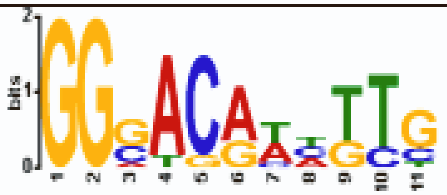	1.8e + 002	64.28	nt no. 10 in S14<br>nt no. 34 in S7<br>nt no. 7 in S8<br>nt no. 11 in S5<br>nt no. 14 in S3<br>nt no. 29 in S13<br>nt no. 3 in S12<br>nt no. 18 in S6<br>nt no. 2 in S1
2	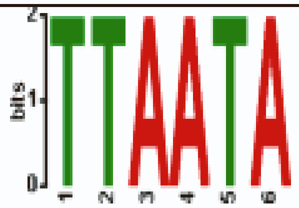	1.9e + 003	14.28	nt no. 6 in S7<br>nt no. 9 in S4
3	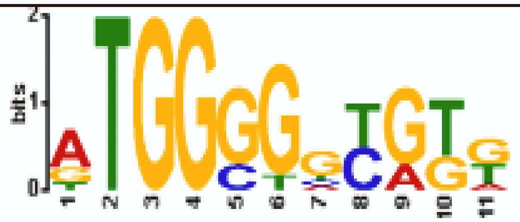	2.5e + 003	57.14	nt no. 23 in S7<br>nt no. 21 in S4<br>nt no. 17 in S11<br>nt no. 22 in S8<br>nt no. 33 in S12<br>nt no. 17 in S1<br>nt no. 7 in S13<br>nt no. 18 in S10
4	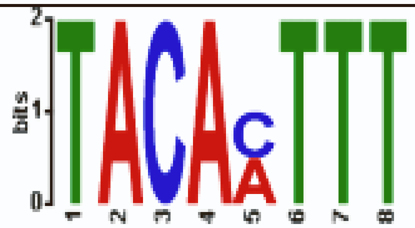	4.3e + 003	14.28	nt no. 2 in S6<br>nt no. 6 in S3
5	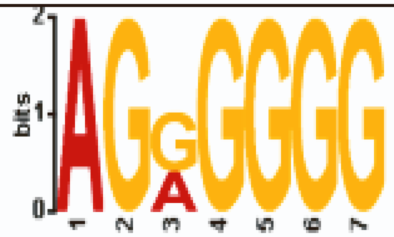	7.1e + 003	35.71	nt no. 5 in S10<br>nt no. 31 in S3<br>nt no. 37 in S1<br>nt no. 26 in S14<br>nt no. 7 in S11

Table S2. Clinical diagnostic performance of S14 aptamer in terms of sensitivity, specificity, positive predictive value, negative predictive value and accuracy.

Statistic	Value
Sensitivity	90.91%
Specificity	98.40%
Positive Predictive Value (*)	93.02%
Negative Predictive Value (*)	97.88%
Accuracy (*)	96.98%

Table S3. Putative overlapping G-quadruplex motifs in the S14 aptamer sequence. Red colored motifs with the highest G-score were used for structure prediction.

S.No.	Start Position	Length	QGRS	G-Score
1	2	31	GGGAGCCTGGGACATAGTGGGGAAAGAGGGG	70
2	2	30	GGGAGCCTGGGACATAGTGGGGAAAGAGGG	70
3	2	30	GGGAGCCTGGGACATAGTGGGGAAAGAGGG	69
4	2	31	GGGAGCCTGGGACATAGTGGGGAAAGAGGGG	69
5	2	40	GGGAGCCTGGGACATAGTGGGGAAAGAGGGGAAGAGTGGG	61
6	2	40	GGGAGCCTGGGACATAGTGGGGAAAGAGGGGAAGAGTGGG	61
7	2	40	GGGAGCCTGGGACATAGTGGGGAAAGAGGGGAAGAGTGGG	61
8	2	40	GGGAGCCTGGGACATAGTGGGGAAAGAGGGGAAGAGTGGG	60
9	2	40	GGGAGCCTGGGACATAGTGGGGAAAGAGGGGAAGAGTGGG	63
10	2	40	GGGAGCCTGGGACATAGTGGGGAAAGAGGGGAAGAGTGGG	63
11	2	40	GGGAGCCTGGGACATAGTGGGGAAAGAGGGGAAGAGTGGG	61
12	2	40	GGGAGCCTGGGACATAGTGGGGAAAGAGGGGAAGAGTGGG	62
**13**	**10**	**32**	**GGGACATAGTGGGGAAAGAGGGGAAGAGTGGG**	**71**
14	10	32	GGGACATAGTGGGGAAAGAGGGGAAGAGTGGG	70

Table S4. Table showing H-bond forming residues and distance between them.

S.No.	H-bonds forming residues	Length of H-bonds
1.	Pro463(B) – G32	2.5 Å
2.	Pro39 (C) – T1	3.4 Å
3.	Asp53 (C) – T1	2.1 Å
4	Asn234(C) - T16	2.3 Å
5.	Thr-376 (C) – G27	2.5 Å

Table S5. Mutant and control aptamer sequences used

Aptamer	Sequence	Bases
S14	TGGGAGCCTGGGACATAGTGGGGAAAGAGGGGAAGAGTGGGTCT	44
S14_M1	TGGGAGCCTGGGACA**C**AGTGGGGAAAGAGGGGAAGAGTGGGTCT	44
S14_M2	TGGGAGCCTG**T**GACATAGTG**T**GGAAAGAG**T**GGAAGAGTG**T**GTCT	44
S14_M3	TGGGAGCCTG**C**GACATAGTG**C**GGAAAGAG**C**GGAAGAGTG**C**GTCT	44
S14_M4	TGGGAGCCTGGGACATAGTG**T**GGAAAGAGGGGAAGAGTG**T**GTCT	44
Control aptamer	TTTTTTTTTTTTTTTTTTTTTTTTTTTTTTTTTTTTTTTTTTTTTTTT	48
G-quad control 1	GCAGAGAGAGAGAAAGTGAGAAGGGCGGGGGGGAGAGGGTGGGGG	45
G-quad control 2	GGGGGTGGTGGGGGGGATAGGGTTGGAGAGAGAGAGAGTGTGT	43

Table S6. Comparative study of available tests for antigen detection for SARS-CoV-2

TEST	TARGET	METHOD	LOD	SENSITIVITY (%)	SPECIFICITY (%)	SAMPLE	READ OUT	REF
RapiGEN	Not specified	LFA	-NA-	62	100	NP/OP Swab	Visual: colored bands	[1]
Liming bio	Not specified	LFA	-NA-	0	90	NP/OP Swab	Visual: colored bands	[1]
Savant	N protein	LFA	-NA-	16.7	100	Throat Swab	Visual: fluorescent bands (under UV light)	[1]
Bioeasy	Not specified	LFA	-NA-	85	100	NP/OP Swab	Visual: fluorescent readers	[1]
DETECTR	E and N gene	LFA with RT-Lamp and cas-12	10,000 copies/mL	95	100	NP/OP Swab	Visual	[2]
SHERLOCK	E, N, ORF1ab gene	LFA with RT-RPA and cas-13a	200 copies/mL	97	100	NP/OP Swab	Visual	[3]
Veritor (BD)	N protein	Chromatographic immunoassay	140 TCID50/swab	83.9	100	NP swab	Veritor Plus Analyzer	[4]
LumiraDx (LumiraDx)	N protein	Fluorescence immunoassay	32 TCID50/swab	97.6	96.6	NP swab	LumiraDx Instrument	[5]
COVID-19 Ag Respi-Strip	N protein	Dipstick LFA	5.4 pM	87.9	77.3	NP Swab	Visual	[6]

BinaxNow (Abbott)	N protein	LFA	22.5 TCID50/ swab	96.7	100	NP Swab	Visual	[7]
Xpert Xpress (Cepheid)	E, N genes	RT-PCR	250 copies/mL	97.8	95.6	NP, MT, OP swab	GeneXpert Dx	[8]
FELUDA	N and S genes	RT-RPA with LFA and cas-9	~ 10 copies	100	97	Saliva	Visual	[9]
Sandwich type COVID-19 Nucleocapsid Protein DNA Aptamers	N protein	Antibody-Aptamer based sandwich ELISA and LFA	1 ng/mL	-NA-	-NA-	sputum, serum, and urine	Visual	[10]
This Study	S protein	ALISA	2nM	91	98	NP/OP swabs	Visual and spectrophotometric	-NA-

LOD: Limit of Detection

LFA: Lateral Flow Assay

NP/OP: Nasopharyngeal/Oropharyngeal

ELISA: Enzyme Linked Immunosorbent Assay

S, E and N: Spike, Envelope and Nucelocapsid

References for Table S6:

1. Weitzel, T., Legarraga, P., Iruretagoyena, M., Pizarro, G., Araos, R., Munita, J.M., and Porte, L. Head-to-head comparison of four antigen-based rapid detection tests for the diagnosis of SARS-CoV-2 in respiratory samples.

2. Broughton, J.P., Deng, X., Yu, G., Fasching, C.L., Servellita, V., Singh, J., Miao, X., Streithorst, J.A., Granados, A., Sotomayor-Gonzalez, A., et al. CRISPR–Cas12-based detection of SARS-CoV-2.

3. Patchsung, M., Jantarug, K., Pattama, A., Aphicho, K., Suraritdechachai, S., Meesawat, P., Sappakhaw, K., Leelahakorn, N., Ruenkam, T., Wongsatit, T., et al. (2020). Clinical validation of a Cas13-based assay for the detection of SARS-CoV-2 RNA. Nat. Biomed. Eng. *4*, 1140–1149.

4. COVID-19 Rapid Antigen Testing | BD Veritor™ Plus System https://bdveritor.bd.com/en-us/rapid-antigen-testing/covid-19.

5. Connected diagnostics and diagnostic-led care solutions | LumiraDx https://www.lumiradx.com/us-en/.

6. Rodriguez-Palacios, A., Zeng, N., Berhane, Y., Vandenberg, O., Mertens, P., De Vos, N., Martiny, D., Jassoy, C., Mirazimi, A., Cuypers, L., et al. (2020). Development and Potential Usefulness of the COVID-19 Ag Respi-Strip Diagnostic Assay in a Pandemic Context. Front. Med. | www.frontiersin.org *1*, 225.

7. Abbott U.S. https://www.abbott.com/corpnewsroom.html.

8. Loeffelholz, M.J., Alland, D., Butler-Wu, S.M., Pandey, U., Perno, C.F., Nava, A., Carroll, K.C., Mostafa, H., Davies, E., Mcewan, A., et al. (2020). Multicenter Evaluation of the Cepheid Xpert Xpress SARS-CoV-2 Test.

9. Azhar, M., Phutela, R., Kumar, M., Ansari, A.H., Rauthan, R., Gulati, S., Sharma, N., Sinha, D., Sharma, S., Singh, S., et al. (2020). Rapid, accurate, nucleobase detection using FnCas9. medRxiv, 2020.09.13.20193581.

10. Zhang, L., Fang, X., Liu, X., Ou, H., Zhang, H., Wang, J., Li, Q., Cheng, H., Zhang, W., and Luo, Z. (2020). Discovery of sandwich type COVID-19 nucleocapsid protein DNA aptamers. Chem. Commun. *56*, 10235–10238.
